# Defined co-cultures of glutamatergic and GABAergic neurons with a mutation in *DISC1* reveal aberrant phenotypes in GABAergic neurons

**DOI:** 10.1186/s12868-024-00858-z

**Published:** 2024-03-04

**Authors:** Johanna Heider, Aaron Stahl, Denise Sperlich, Sophia-Marie Hartmann, Sabrina Vogel, Ricarda Breitmeyer, Markus Templin, Hansjürgen Volkmer

**Affiliations:** Department of Pharma and Biotech, NMI Natural and Medical Sciences Institute at the University of Tübingen, 72770 Reutlingen, Germany

**Keywords:** DISC1, E/I imbalance, GABAergic neurons, Glutamatergic neurons, iPSC, Co-culture

## Abstract

**Background:**

Mutations in the gene *DISC1* are associated with increased risk for schizophrenia, bipolar disorder and major depression. The study of mutated DISC1 represents a well-known and comprehensively characterized approach to understand neuropsychiatric disease mechanisms. However, previous studies have mainly used animal models or rather heterogeneous populations of iPSC-derived neurons, generated by undirected differentiation, to study the effects of DISC1 disruption. Since major hypotheses to explain neurodevelopmental, psychiatric disorders rely on altered neuronal connectivity observed in patients, an ideal iPSC-based model requires accurate representation of the structure and complexity of neuronal circuitries. In this study, we made use of an isogenic cell line with a mutation in *DISC1* to study neuronal synaptic phenotypes in a culture system comprising a defined ratio of NGN2 and ASCL1/DLX2 (AD2)-transduced neurons, enriched for glutamatergic and GABAergic neurons, respectively, to mimic properties of the cortical microcircuitry.

**Results:**

In heterozygous *DISC1* mutant neurons, we replicated the expected phenotypes including altered neural progenitor proliferation as well as neurite outgrowth, deregulated DISC1-associated signaling pathways, and reduced synaptic densities in cultures composed of glutamatergic neurons. Cultures comprising a defined ratio of NGN2 and AD2 neurons then revealed considerably increased GABAergic synapse densities, which have not been observed in any iPSC-derived model so far. Increased inhibitory synapse densities could be associated with an increased efficiency of GABAergic differentiation, which we observed in AD2-transduced cultures of mutant neurons. Additionally, we found increased neuronal activity in GABAergic neurons through calcium imaging while the activity pattern of glutamatergic neurons remained unchanged.

**Conclusions:**

In conclusion, our results demonstrate phenotypic differences in a co-culture comprising a defined ratio of *DISC1* mutant NGN2 and AD2 neurons, as compared to culture models comprising only one neuronal cell type. Altered synapse numbers and neuronal activity imply that DISC1 impacts the excitatory/inhibitory balance in NGN2/AD2 co-cultures, mainly through increased GABAergic input.

**Supplementary Information:**

The online version contains supplementary material available at 10.1186/s12868-024-00858-z.

## Background

The human cortex is primarily composed of excitatory and inhibitory neurons organized within the cortical local microcircuitry comprising inhibitory GABAergic interneurons to control excitatory, glutamatergic principal neuron output [1]. GABAergic control may contribute to the synchronization of neuronal networks which is important for proper cognition, memory, and learning [2]. Imbalance of excitation and inhibition (E/I imbalance) in the local microcircuitry is considered as one factor to contribute to a variety of neurodevelopmental disorders such as autism and schizophrenia. Therefore, the study of pathological mechanisms addressing E/I imbalance in neurodevelopmental disorders requires human models enabling interaction of excitatory and inhibitory neurons with each other.

With the possibility to generate induced pluripotent stem cells (iPSCs) and to differentiate these cells into specific neuronal phenotypes, an opportunity developed to create co-culture models comprising excitatory and inhibitory neurons harbouring a human genetic background. Different approaches have been proposed in the past to establish such models. One is based on differentiation with exogenously added factors providing a less defined network of excitatory and inhibitory neurons [3, 4]. Another strategy relies on directed differentiation of either excitatory or inhibitory neurons by endogenous expression of inducible transcription factors NGN2 or ASCL1/DLX2 (AD2), respectively [5, 6]. Co-culture models using the latter protocol enable the study of neuronal networks [7–9] after combination of different cell types at highly defined ratios that are more suitable to study E/I imbalance in neurodevelopmental disorders. Moreover, it has been reported that upon co-culture with NGN2 neurons, AD2 neurons show improved morphological and functional maturity [10]. As an example, a recent study made use of such a defined network of induced glutamatergic and GABAergic neurons to study functional and morphological consequences of loss-of-function mutations in the SCZ risk gene *SETD1*A on single-cell and network level [11].

The *DISC1* gene (Disrupted-in-schizophrenia 1) was originally identified in a large Scottish family with a balanced chromosomal t (1;11) translocation [12]. Mutation of *DISC1* predisposes to severe psychiatric disorders including schizophrenia, major depressive disorder, or bipolar disorder. The DISC1 protein is a scaffolding protein interacting with at least 16 other proteins [13] contributing to a variety of cellular or more specifically synaptic functions such as mRNA, synaptic vesicle and mitochondrial trafficking, as well as dendritic spine stabilization and synaptic plasticity.

To date, *DISC1* mutations were used as a model for psychiatric disorders and E/I imbalance, mainly in rodent models. A critical contribution of GABAergic interneurons in rodent models is well established [14, 15]. On the other hand, human iPSC-derived neurons were applied to study DISC1 functions in human neurons after growth factor induction [3, 16–19]. However, the crucial contribution of interneurons was not reported in any of these models.

Our study aims at the phenotypic evaluation of an iPSC-based co-culture system comprising NGN2-transduced cultures, enriched for glutamatergic neurons, co-cultured with ASCL1/DLX2 (AD2)-transduced cultures, enriched for GABAergic neurons, as a model for excitatory-inhibitory neuron interaction. An isogenic pair of iPSC lines, one with a heterozygous *DISC1* mutation in exon 2 (*DISC1* ±), were comprehensively characterized to ensure that the *DISC1* mutation line shows representative phenotypical features of DISC1 deficiency, which have previously been described. When cultured separately, excitatory synapses were reduced in NGN2 neurons, whereas inhibitory synapses in AD2 neurons remained unaffected. In a defined co-culture system of NGN2 and AD2 neurons, however, the E/I balance was shifted due to an increase of inhibitory synapses. In parallel, the activity of AD2 neurons was increased.

## Methods

### iPSC line generation and maintenance

As described in Heider et al. (2021), the *DISC1* ± iPSC line (TMOi001-A-5) was generated by the introduction of a heterozygous frameshift mutation in exon 2 by CRISPR-Cas9 gene-editing of a healthy iPSC line (TMOi001-A). The isogenic parental line was obtained from Thermo Fisher Scientific (Gibco^™^ episomal hiPSC line #A18945). iPSC lines used in this study are fully characterized, chromosomally intact and express stem cell markers on DNA and RNA level. iPSC were maintained in mTeSR Plus medium (STEMCELL Technologies, #100-0276) on Matrigel (Corning, # 354277)-coated 6-well plates. The cells were enzymatically passaged for single-cell seeding using Accutase (Sigma-Aldrich, # A6964).

### NPC differentiation and maintenance

iPSC were differentiated into neural progenitor cells using the STEMdiff^™^ SMADi Neural Induction Kit, following the embryoid body (EB) protocol (STEMCELL Technologies #08581). Briefly, iPSC were seeded at a density of 2 × 10^6 per well into AggreWell^™^ 800 plates in STEMdiff^™^ SMADi Neural Induction medium, pretreated with Anti-Adherence Rinsing Solution (STEMCELL Technologies #07010). Daily medium changes were performed for seven days to allow the formation of EBs. Afterwards, EBs were harvested using 37 µm reversible strainers (STEMCELL Technologies #27215) and plated onto poly-l-ornithine/Laminin-coated 6-well plates. For coating, plates were treated with 20% poly-l-ornithine (PLO, Sigma-Aldrich #P4957) in DPBS for 2 h at RT, then washed with DPBS and DMEM/F12, followed by incubation with Laminin (10 µg/ml, Sigma-Aldrich #L2020) in DMEM/F12 overnight at 37 °C. EBs were cultivated for seven days with daily medium changes in STEMdiff^™^ SMADi Neural Induction medium. Afterwards, neural rosettes were selected with STEMdiff^™^ Neural Rosette Selection Reagent (STEMCELL Technologies #05832) for 1.5 h at 37 °C and plated on PLO/Laminin-coated 6-well plates in STEMdiff^™^ Neural Progenitor medium (STEMCELL Technologies #05833). NPC were maintained in Neural progenitor medium for cultivation and enzymatically passaged using Accutase. NPC were used for neuronal differentiations until passage 10.

### Neurite outgrowth

For neurite outgrowth assays, NPC were seeded at a density of 3 × 10^4 cells/cm^2^ on Matrigel-coated 96-well plates in Neural Progenitor medium. For coating, hESC-qualified Matrigel^®^ Matrix (Corning #354277) was diluted 1:100 in ice-cold DMEM/F12 according to the manufacturer’s instructions and incubated for 30 min at 37 °C. On the following day, the medium was changed to 50% DMEM/F12 (Thermo Fisher Scientific #10565018) + 50% Neurobasal^™^ Plus medium (Thermo Fisher Scientific #A3582901), supplemented with 1xB27 Plus supplement (Thermo Fisher Scientific #A3582801), 1 × N2 supplement (Thermo Fisher Scientific #17502048), 5 µg/ml insulin (Sigma Aldrich), 1 × GlutaMax (Thermo Fisher Scientific #35050061), 1 × non-essential amino acids (Thermo Fisher Scientific #11140035), 1 × Penicillin/Streptomycin (Thermo Fisher Scientific #15070063) and 0.5 mM of β-Mercaptoethanol (Carl Roth #4227). Outgrowing neurites were imaged every 4 h using IncuCyte^®^ (Sartorius AG, Göttingen, Germany). Mean neurite length normalized to cell body area per image was determined with the IncuCyte NeuroTrack Analyzer tool. Data were analyzed from 4 independent experiments with at least 3 wells (4 images/well; ~ 800 cells per image) per replicate. The four images of one well were averaged to give yield to 1 n.

### NPC proliferation and cell death

NPC were seeded at a density of 3 × 10^4^/cm^2^ on Matrigel-coated 96-well plates in Neural Progenitor medium. After cell attachment, NPC were stained with 5 µM of SYTOX^™^ Orange Nucleic Acid Stain (Thermo Fisher Scientific #S11368) for 30 min at 37 °C to label cells with compromised membranes. Afterwards, cells were washed DMEM/F12 and medium was changed back to Neural Progenitor medium. Cells were imaged in brightfield mode and dead cells in fluorescence mode for 3 days every 4 h using IncuCyte^®^. NPC proliferation and cell death were analyzed using the IncuCyte^®^ Basic Analyzer. Confluence was normalized to t = 0 at every time point and cell death numbers were normalized to the respective confluence at every time point. Data were analyzed from 3 independent experiments with at least 5 wells imaged per replicate.

### Neuronal differentiation

For neuronal differentiations, NPC were seeded at a density of 4.29 × 10^4^/cm^2^ on PLO/Laminin-coated 12-well plates in Neural Progenitor medium (DIV1). On DIV0, cells were transduced with pLV-TetO-hNGN2-eGFP-Puro + FUdeltaGW-rtTA (Addgene #79823, #19780) for excitatory neuron differentiation and TetO-Ascl1-puro + DLX2-hygro + FUdeltaGW-rtTA (Addgene #97329, #97330, #19780) for inhibitory neuron differentiation in separate wells according to published protocols [5, 6]. The approximate final concentration of viral particles used is 2 × 10^8^ particles/ml. 24 h after viral transduction (DIV1), medium was changed to Neural progenitor medium containing 1 µg/ml of Doxycycline (Sigma-Aldrich #D9891) to induce tetracycline-dependent transgene expression. 24 h after induction (DIV2), antibiotics were added to the medium to select for transduced cells (2 µg/ml puromycine (Thermo Fisher Scientific #A1113803) for NGN2-transduced cells, 2 µg/ml of puromycine + 250 µg/ml of hygromycin (Carl Roth #CP12.1) for ASCL1/DLX2-transduced cells). On DIV3, excitatory (NGN2) and inhibitory (ASCL1 + DLX2) neurons were detached with Accutase and seeded separately, or co-cultures at a ratio of 80:20 (total neuronal density was 1.25 × 10^5^ neurons per cm^2^ for both culture types) on PLO/Laminin-coated plates. Neuronal medium consisted of Neurobasal Plus medium, supplemented with 1 × B27 Plus supplement, 1 × N2 supplement, 1 µg/ml of laminin, 20 ng/ml of GDNF (Peprotech #450-10), 20 ng/ml of BDNF (Peprotech #450–02), 35 µg/ml of L-Ascorbic Acid (Sigma-Aldrich #A2078), 1 × Penicillin/Streptomycin and 1 µg/ml of Doxycycline. After neuronal attachment, murine primary astrocytes were added to NGN2, AD2, or NGN2/AD2 cultures at a density of 3.125 × 10^4^/cm^2^. 50% medium changes with neuronal medium (without Doxycycline) were performed until DIV24, when neuronal medium was again once supplemented with 1 µg/ml Doxycycline to support neuronal maturation at the final stages of the differentiation process until DIV28.

### Immunocytochemistry

Cells were fixed with 4% PFA in PBS for 15 min at RT and washed 3 × with PBS. For blocking and permeabilization, cells were incubated with blocking solution (1 × blocking reagent BMB (Roche #11,112,589,001) + 0.1% Triton X-100 (Carl Roth #3051) in PBS) for 30 min at RT. Primary antibodies were diluted in blocking solution and incubated overnight at 4 °C. Next, cells were washed 3 × with PBS and secondary antibodies, diluted in blocking solution, were applied for 2 h at RT. Cells were again washed 3 × with PBS and stored in PBS.

### Imaging and analysis of DISC1 protein expression

Imaging of DISC1 protein expression in iPSC and NPC was performed using the spinning disk confocal microscope Cell Observer SD (Zeiss, Oberkochen, Germany) with a plan-apochromatic 20 × objective. From 3 independent experiments, 3–5 fields of view (containing multiple cells) were imaged per well. 2 wells were imaged for each condition in every biological replicate. From each microscopic field, DISC1 mean fluorescence intensity of 4 individual cells was measured by hand-drawn regions of interest (ROI) using the software ZEN 3.0 (Zeiss). Background intensities were measured per microscopic field and subtracted from DISC1 intensity values, which were afterwards normalized to WT mean within individual biological replicates. Data points in the graphs represent the average fluorescence intensity of multiple cells measured from a single microscopic field.

### Western blot

iPSC were dissociated by Accutase treatment, washed with PBS and lysed on ice in RIPA buffer (Thermo Fisher Scientific #89,900), supplemented with protease inhibitor mix (Serva # 39,102), for 30 min. Samples were vortexed every 10 min and subsequently centrifuged at 14000×g for 20 min at 4 °C. Protein concentration of the resulting supernatant was determined with a Pierce BCA protein assay (Thermo Fisher Scientifc #23225), according to the manufacturer’s instructions. 10 µg of protein per sample were mixed with 1 × NuPAGE LDS sample buffer (Thermo Fisher Scientific #NP0008) and 1 × NuPAGE sample reducing agent (Thermo Fisher Scientific #NP0004) and heated for 10 min at 70 °C. Samples and PageRuler plus prestained protein ladder (Thermo Fisher Scientific #26,619) were loaded onto NuPAGE bis–tris gradient gels (Thermo Fisher Scientific #NP0321BOX). Gels were run in 1 × NuPAGE MOPS SDS running buffer (Thermo Fisher Scientific #NP0001), supplemented with NuPAGE Antioxidant (Thermo Fisher Scientifc #NP0005) at 200 V. Semi-dry blotting on PVDF transfer membranes (Thermo Fisher Scientific #88,518), soaked in 1 × NuPAGE transfer buffer (Thermo Fisher Scientific #NP0006), was performed for 80 min (2 mA/cm^2^, 250 V). Membranes were blocked with 1 × TBST buffer + 5% BSA for 60 min and primary antibodies detecting N-terminal DISC1 (Merck #ABN469, 1:500) and beta-actin (Sigma-Aldrich #A5316, 1:5000) were applied separately overnight at 4 °C in 1 × TBST buffer + 5% BSA. Membranes were washed 3 × 5 min with 1 × TBST and secondary antibodies were applied for 1 h at RT in 1 × TBST buffer + 5% BSA. Membranes were again washed three times and imaged with the Typhoon biomolecular imaging system (Cytiva, USA). Resulting bands were analyzed with the software Image Lab (Bio-Rad, USA). DISC1 band volume was normalized to beta-actin.

### Quantification of GABA-positive neurons

DIV28 NGN2 and AD2 neurons were stained against MAP2 and GABA and z-stack confocal images were acquired with the spinning disk confocal microscope, using a 40 × objective (Zeiss, Oberkochen Germany). Images were acquired from two wells per clone per differentiation and from two independent neuronal differentiations. 5 images were acquired per well. Orthogonal maximum intensity projections of the images were created using the software Zen 3.0 (Zeiss). Using Fiji (Image J), up to 10 MAP2-positive somata per image were randomly selected from images showing only the MAP2 channel, with the multi point tool. The selections were saved as ROIs and loaded onto the image containing only the channel with GABA staining to quantify how many of the pre-selected somata were GABA-positive. Percentages of GABA-positive neurons were calculated per image.

### Imaging and analysis of synaptic marker expression

Imaging of synaptic marker expression in DIV28 neurons was performed using the spinning disk confocal microscope Cell Observer SD (Zeiss, Oberkochen, Germany) with a plan-apochromatic 63 × oil immersion objective. Images were acquired from 3 to 5 independent neuronal differentiations. 2 wells were imaged for each condition in each replicate. Within each well, 10 fields of view (z-stack images) with comparable MAP2-network density were acquired.

Analysis of synaptic densities was performed using IMARIS Bitplane 10.0.0 (Oxford Instruments, Abigdon, UK) as show in Additional file 1: Figure S1. For every individual microscopic field, a 3D mask of the MAP2 signal was created used to filter all synaptic signals lying within the MAP2 volume. Synapse numbers within the MAP2 volume were then quantified using the spot detection tool. Intensity thresholds for spot detection and spot size limits were kept constant within individual biological replicates for each synaptic marker. To analyze the co-localization of pre- and postsynaptic markers, the IMARIS XTension plugin ‘Spots Colocalize’ was used. A maximum distance of 0.2 µm between pre- and postsynaptic spots was defined, corresponding to the resolution limit of the Cell Observer SD microscope.

To distinguish between synapses on either NGN2 or AD2 neurons, a 3D mask of the GFP signal of NGN2 neurons was created. Synaptic channels were again filtered to exclude spots localized outside the GFP mask. Quantification of synapse densities and co-localization analysis was performed as described above. Synapse densities on AD2 neurons were derived by subtraction of synapse numbers on NGN2 neurons from the total synapse number on the MAP2 network. To account for differences in neuronal network density, all synapse numbers were normalized to the volume of either the MAP2 mask (all neurons), GFP mask (NGN2 neurons), or the difference of both (AD2 neurons).

Synapse densities from individual fields of view within a single well were averaged and normalized to the WT synaptic marker densities.

### Calcium imaging

DIV28 neurons were stained with 2 µM of Calbryte^™^ 590 AM (AAT Bioquest #20,700) diluted in neuronal medium for 30 min at 37 °C, 5% CO_2_. Afterwards, cells were washed with DMEM/F12 and medium was replaced by neuronal medium. Before imaging, the neurons were incubated for 15 min at 37 °C, allowing them to recover. To record spontaneous single-cell calcium activity, neurons were imaged at 37 °C, 5% CO_2_ using the spinning disc confocal microscope Cell Observer SD. 3 min recordings of multiple cells within a microscopic field were obtained from 4 independent neuronal differentiations. For each differentiation, 4 wells with 2 videos per well were recorded. For each microscopic field, a snapshot of the Calbryte^™^ signal (staining all neurons) and GFP signal of NGN2 neurons was obtained to allow for cell-type specific analysis. Somatic calcium signals were measured from individual neurons by hand-drawing ROI with Fiji and extracting the mean fluorescence intensity over time. A minimum of 3 spontaneously active neurons (min. one calcium peak per recording period) of both subtypes (NGN2, AD2) were analyzed per recording. Extracted traces were further analyzed with the Origin 2015G Peak Analyzer module (OriginLab Corporation). Traces were normalized to baseline, peak detection threshold and minimum peak distance were kept constant across all replicates. Peak parameters (AUC, FHWM, Amplitude) were averaged across all peaks of a single neuron. Data points in the graphs are normalized to the WT mean and represent the mean of multiple cells per well.

### DigiWest

#### Lysis and protein quantification

For DigiWest cultures without astrocytes were used. 40 µl of RIPA lysis buffer (Thermo Fisher Scientific #89900) supplemented with cOmplete^™^ mini protease inhibitor (Roche # 11836153,001) was added to cell pellets on ice. Cells were lysed for 30 min at 4 °C. Protein quantification was performed using in-gel staining. 1 µl of each original cell lysate was diluted 1:10 in lysis buffer. 10 µl of the respective aliquots were loaded onto a NuPAGE 4–12% Bis–Tris precast gel (Thermo Fisher Scientific # NP0336BOX) and run according to the manufacturer´s instructions. The gel was washed with water and proteins were stained with BlueBandit (VWR # K217-1L) for 1 h. The gel was de-stained over night with ddH_2_O before detection at a LI-COR (LI-COR, Bad Homburg, Germany) instrument. Analysis and protein quantification was performed using ImageStudio (LI-COR).

#### DigiWest protein profiling

DigiWest was performed as published [20]. A graphical representation of the workflow can be found in Additional file 1: Figure S2. In brief, 12 μg of cellular protein was loaded on an SDS- polyacrylamide gel and size-separated using the commercial NuPAGE system (Life Technologies). Size-separated proteins were blotted onto a PVDF membrane and biotinylated on the membrane using NHS-PEG12-Biotin (50 µM) in PBST for 1 h. After washing with PBST and drying of the membrane, the individual samples lanes were cut into 96 strips of 0.5 mm width using an automated cutting plotter (Silhouette America, West Orem, UT, USA) each corresponding to a defined molecular mass fraction. Each of the strips was placed in one well of a 96-well plate and 10 µl elution buffer (8 M urea, 1% Triton-X100 in 100 mM Tris–HCl pH 9.5) was added. The eluted proteins were diluted with 90 μl of dilution buffer (5% BSA in PBS, 0.02% sodium azide, 0.05% Tween-20) and each of the protein fractions was incubated with 1 distinct magnetic color-coded bead population (Luminex, Austin, USA) coated with neutravidin. The biotinylated proteins bind to the neutravidin beads such that each bead color represents proteins of one specific molecular mass fraction. All 96 protein loaded bead populations were mixed resulting in reconstitution of the original lane. Aliquots of the DigiWest bead-mixes (about 1/200th per well) were added to 96 well plates containing 50 µl assay buffer (Blocking Reagent for ELISA (Roche #11112589,001) supplemented with 0.2% milk powder, 0.05% Tween-20 and 0.02% sodium azide). Beads were briefly incubated in assay buffer and buffer was discarded. Primary antibodies (complete list can be found in Additional file 2: Table S1) were diluted in assay buffer and 30 μl were added per well. After overnight incubation at 15 °C, the bead-mixes were washed twice with PBST and species-specific PE-labelled (Phycoerythrin) secondary antibodies (Dianova, Hamburg, Germany) were added and incubated for 1 h at 23 °C. Beads were washed twice with PBST prior to readout on a Luminex FlexMAP 3D.

#### Data analysis

For quantification of the antibody-specific signals, an Excel-based analysis tool was employed [20] that automatically identifies peaks of appropriate molecular mass and calculates the peak area (reported as accumulated fluorescence intensity = AFI). Signal intensity was normalized to the total amount of protein loaded onto one lane. The software package MEV 4.9.0 was used for heatmap generation [21] along with GraphPad Prism (Version 9.0.0). Wilcoxon tests were performed to compare protein expression in WT and *DISC1* ± neurons, and proteins with significantly different expression levels (p < 0.05) were plotted in the heatmap.

### Statistical analysis

Statistical analysis was performed using Graph Pad Prism (Version 9.0.0). For pairwise comparisons, unpaired Mann–Whitney U tests were performed. For the analysis of neurite outgrowth, NPC proliferation and cell death, Two-way ANOVA and Šídák's multiple comparisons test was used. For DigiWest protein profiling analysis the Wilcoxon rank-sum test was employed. P-values were assigned as follows: * = p < 0.05, ** = p < 0.01, *** = p < 0.001, **** = p < 0.0001.

## Results

### Phenotypes of NPC with a heterozygous *DISC1* mutation

**Fig. 1:**
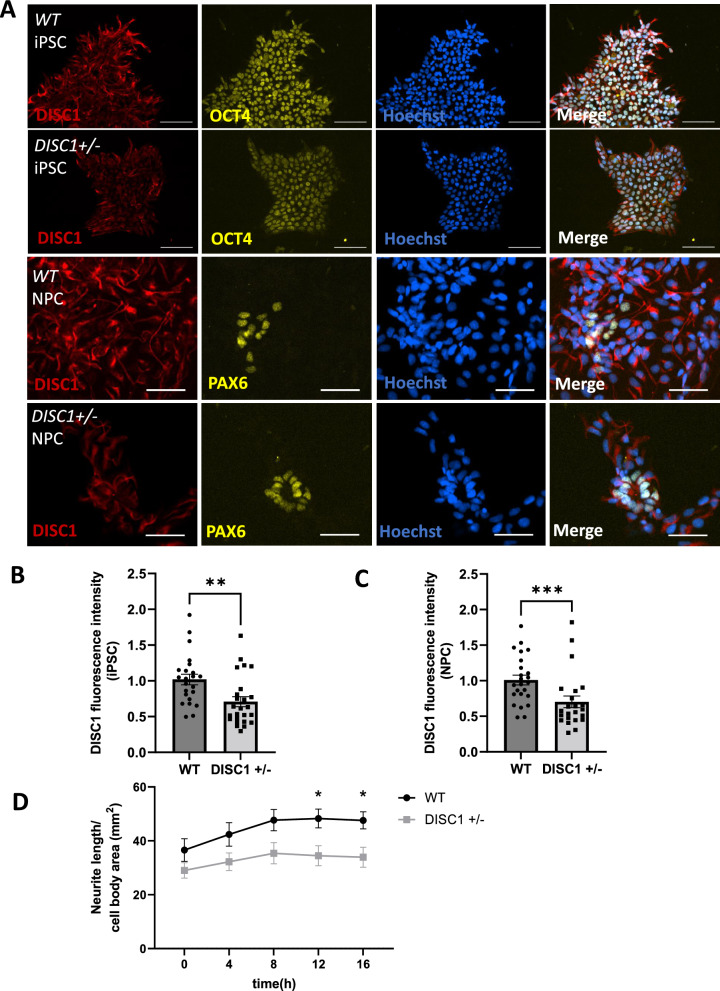
Neurite outgrowth of differentiating *DISC1* ± NPCs. **A** DISC1 immunocytochemistry of WT and *DISC1* ± iPSC and NPC. iPSC are OCT4 positive (Scale bars: 100 µm), and subpopulations of NPC are PAX6 positive (Scale bars: 50 µm). **B**, **C** Quantification of DISC1 fluorescence intensity of WT and *DISC1* ± iPSC (WT = 1 ± 0.07, *DISC1* ±  = 0.7 ± 0.07) and NPC (WT = 1 ± 0.07, *DISC1* ±  = 0.7 ± 0.08). Data points represent averaged fluorescence intensities of multiple cells within a single microscopic field. Data were obtained from three independent experiments. iPSC: WT n = 23, *DISC1* ± n = 25, Mann–Whitney U test, two-tailed, p = 0.002. NPC: WT n = 24, *DISC1* ± n = 23, Mann–Whitney U test, two-tailed, p = 0.0008. **D** Neurite outgrowth assay of differentiating NPC. Neurite length per cell body area was measured every 4 h over a period of 16 h and was significantly reduced in *DISC1* ± cells at t = 12 h and t = 16 h (0 h: WT = 36.6 ± 4.2, *DISC1* ±  = 29 ± 2.9; 4h: WT = 42.4 ± 4.4, *DISC1* ±  = 32.2 ± 3.3; 8h: WT = 47.7 ± 3.9, *DISC1* ±  = 35.4 ± 3.9; 12 h: WT = 48.3 ± 3.5, *DISC1* ±  = 34.5 ± 3.7; 16h: WT = 47.6 ± 3.2, *DISC1* ±  = 33.9 ± 3.7). Data were obtained from 4 independent experiments with at least 3 wells (4 images each) imaged per condition and replicate. WT n = 24, *DISC1* ± n = 24 (for each timepoint). (2way ANOVA with Šídák's multiple comparisons test, t = 12 h p = 0.0423, t = 16 h p = 0.0463). Error bars: s.e.m

For the analysis of a neuronal co-culture we chose *DISC1* mutation as a reliable and extensively evaluated model for psychiatric disorders [16, 17, 19, 22–24]. To this end, we generated isogenic iPSC after introducing a heterozygous mutation into exon 2 of *DISC1*. Cells were fully characterized, and off-target mutations excluded, as described previously [25]. Theoretically, the frame shift mutation allows for the translation of a truncated version of full length DISC1 encompassing the first 88 amino acid residues before translation becomes terminated at a STOP codon after additional 39 irregular amino acid residues [25, 26]. The parental wild-type line and the mutated heterozygous line (*DISC1* ±) were differentiated into neuronal progenitor cells (NPC) and submitted to immunocytochemistry analysis to evaluate DISC1 expression using an antibody specific for the COOH terminus of DISC1 (Fig. 1A). Quantification of the fluorescence signals revealed a partial reduction of DISC1 staining both in iPSC and in NPC in accordance with a heterozygous mutation (Fig. 1B, C). In addition, Western blotting using an antibody directed against the N-terminus of the DISC1 protein was performed in iPSC (Additional file 1: Figure S3). A band was detected at $\sim$ 95 kDa, corresponding to the L/Lv-isoform of the DISC1 protein (Additional file 1: Figure S3A). Quantification of the $\sim$ 95 kDa band showed a partial reduction of DISC1 protein level in the *DISC1* ± clone (WT = 1 $\pm$ 0.16, *DISC1* ±  = 0.77 $\pm$ 0.13, Additional file 1: Figure S3B).

Next, we performed a set of experiments to replicate previous observations and to assure a representative phenotype in our *DISC1* ± line. Since DISC1 was suggested to play a role in neuronal proliferation [27], we tested NPC proliferation over time. Proliferation was increased for the *DISC1* ± clone while analysis of cell death showed no apparent differences between the isogenic clones (Additional file 1: Figure S4). NPC were exposed to growth factors to induce neurite extension to be quantified with an IncuCyte ^®^ device for unbiased, fully automated neurite outgrowth quantification. In accordance with previous studies, a reduction of neurite length in *DISC1* ± neurons was observed (Fig. 1D, [24, 28]).

### Synapse densities in *DISC1* ± NGN2 and AD2 neurons cultured separately

**Fig. 2:**
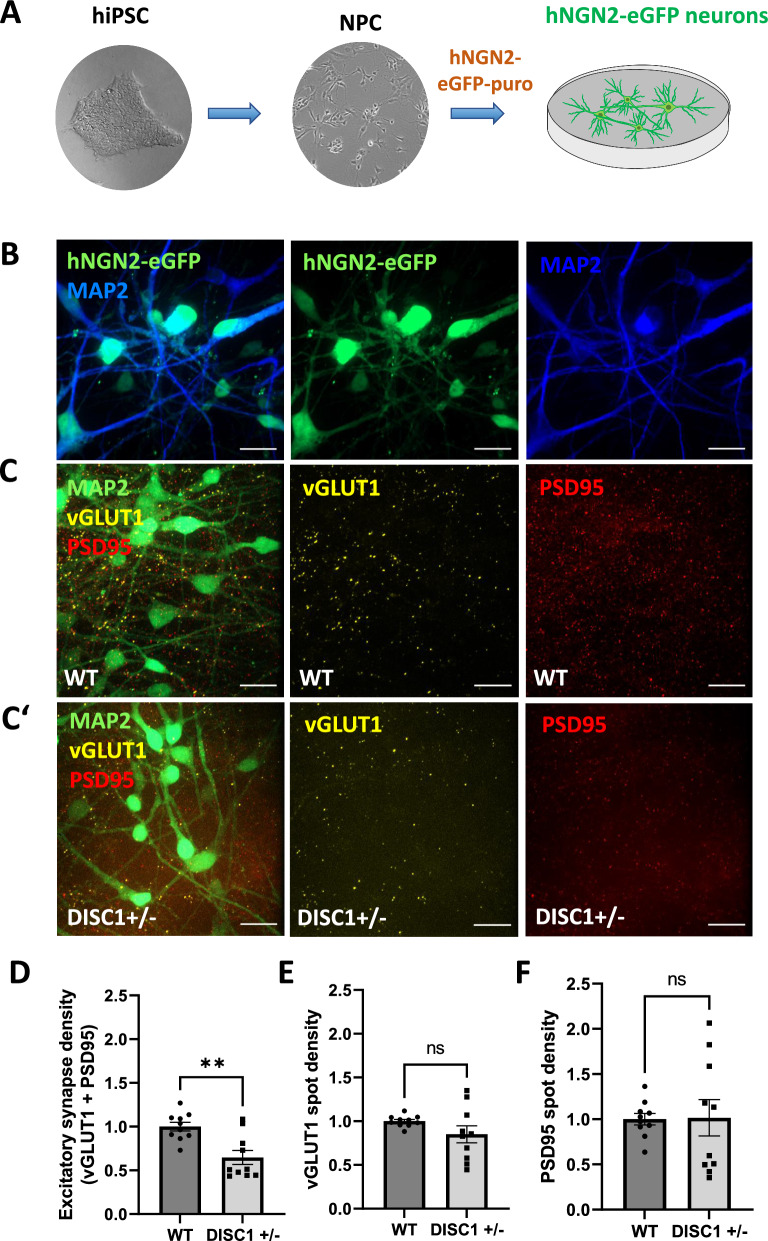
Excitatory synapse density in *DISC1* ± NGN2 neurons. **A** Scheme of NGN2 neuron differentiation. iPSC were first differentiated into NPC and subsequently transduced with doxycycline-inducible hNGN2-eGFP. **B** Fluorescence microscopy for microtubule-associated protein 2 (MAP2) immunostaining and detection of eGFP expression in NGN2 neurons. Scale bars: 20 µm. **C–C’** Expression of MAP2, presynaptic marker vesicular glutamate transporter 1 (vGLUT1) and postsynaptic marker postsynaptic density protein 95 (PSD95) in WT (**C**) or *DISC1* ± (**C’**) NGN2 neurons. Scale bars: 20 µm. **D–F** Quantification of excitatory synapse marker densities in *DISC1* ± NGN2 neurons; WT n = 10, *DISC1* ± n = 10. Data were collected from five independent differentiations. **D** Excitatory synapse densities defined as apposition of presynaptic vGLUT1 and postsynaptic PSD95 clusters: WT = 1 ± 0.05, *DISC1* ±  = 0.65 ± 0.08, Mann–Whitney U test, two-tailed, p = 0.0039. **E** Presynaptic vGLUT1 cluster densities: WT = 1 ± 0.02, *DISC1* ±  = 0.85 ± 0.09, Mann–Whitney U test, two-tailed, p = 0.1431. **F** Postsynaptic PSD95 cluster densities: WT = 1 ± 0.06, *DISC1* ±  = 1.02 $\pm$ 0.2, Mann–Whitney U test, two-tailed, p = 0.7959). Error bars: s.e.m

For further assessment of known DISC1 phenotypes, lentiviral transduction and overexpression of NGN2 in NPC was applied to generate populations enriched for glutamatergic, excitatory neurons [6] (Fig. 2A). At DIV 28, NGN2 transduced NPC gave rise to MAP2-positive neurons expressing co-transduced eGFP (Fig. 2B) as well as glutamatergic markers vGLUT1 and PSD95, which were partially co-localizing, indicating the formation of structurally intact glutamatergic synapses (Fig. 2C, C’, Additional file1: figure S5A). The MAP2 signal was used as a mask to identify synapse marker densities on neuronal cells. Quantification of excitatory synapse densities, as defined by apposition of presynaptic marker vGLUT1 with postsynaptic marker PSD95, revealed a significant decrease of glutamatergic synapses in *DISC1* ± NGN2 neurons (Fig. 2D). In contrast, quantification of vGLUT1 and PSD95 clusters, separately, did not show significant differences (Fig. 2E, F). Thus, glutamatergic synapses are mostly affected in *DISC1* heterozygous NGN2 neurons in agreement with a previous report [3].

**Fig. 3:**
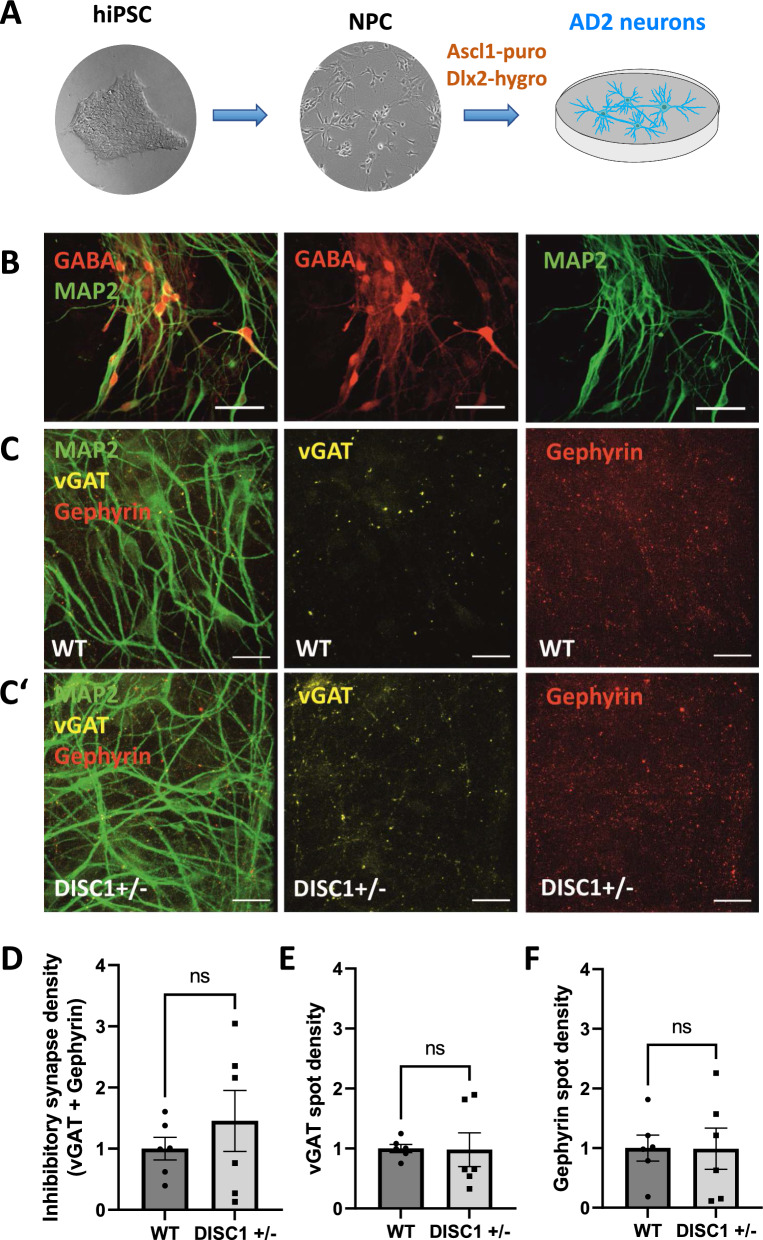
Inhibitory synapse density in *DISC1* ± AD2 neurons. **A** Scheme of AD2 neuron differentiation. iPSC were first differentiated into NPC and subsequently transduced with doxycycline-inducible ASCL1 and DLX2. **B** Fluorescence microscopy for microtubule-associated protein 2 (MAP2) and GABA immunostaining in AD2 neurons. Scale bars: 50 µm. **C–C’** Expression of MAP2, presynaptic marker vesicular GABA transporter (vGAT) and postsynaptic marker Gephyrin in WT (**C**) or *DISC1* ± (**C’**) AD2 neurons. Scale bars: 20 µm. **D-F** Quantification of GABAergic synapse marker densities in *DISC1* ± AD2 neurons; WT n = 6, *DISC1* ± n = 6. Data were obtained from three independent differentiations. **D** Inhibitory synapse densities (co-localization of vGAT + gephyrin): WT = 1 ± 0.18, DISC1 ±  = 1.45 ± 0.5, Mann–Whitney U test, two-tailed, p = 0.8182. **E** Presynaptic vGAT cluster densities: WT = 1 ± 0.07, DISC1 ±  = 0.98 ± 0.28, Mann–Whitney U test, two-tailed, p = 0.3939. **F** Postsynaptic gephyrin cluster densities: WT = 1 ± 0.22, DISC1 ±  = 0.99 ± 0.35, Mann–Whitney U test, two-tailed, p = 0.9372. Error bars: s.e.m

Transduction of NPC with ASCL1/DLX2 (AD2) was used to generate neuronal cultures enriched for GABAergic, inhibitory neurons [5] (Fig. 3A). Immunostaining showed GABA-specific signals on MAP2-positive neurons (Fig. 3B). In a previous report, AD2-transduced iPSC yielded cultures comprised of ~ 90% GABA-positive neurons 5 weeks after transduction [10]. To evaluate purity of NPC-derived AD2 cultures, the percentage of MAP2/GABA-double-positive neurons was quantified in WT and *DISC1* ± NGN2 and AD2 cultures. There were significantly more GABA-positive cells in cultures containing AD2 neurons as compared to NGN2 cultures, where only very few GABA-positive cells were observed (WT AD2= $\sim$ 42%, WT NGN2 =  ~ 2%, *DISC1* ± AD2 =  ~ 61%, *DISC1* ± NGN2 =  ~ 5%, Additional file 1: Figure S6A, B), which is in accordance with previous work [29]. Therefore, we concluded that while NGN2-transduced neurons seem to result in a more homogenous cell population, our AD2-transduced cultures might contain additional neuronal cell types or more immature, still unspecified types of neurons.

Interestingly, when directly comparing WT and *DISC1* ± AD2 cultures, a significant increase of the percentage of GABA-positive neurons was observed in mutant cultures (Additonal file 1: Figure S6C), suggesting enhanced differentiation efficiency towards the GABAergic lineage. WT and *DISC1* ± cells expressed partially co-localizing GABAergic markers vGAT and gephyrin, indicating the presence of inhibitory synapses (Fig. 3C, C’, Additional file 1: Figure S5B). Quantification of GABAergic marker densities revealed no difference on WT and *DISC1* ± neurons neither after evaluation of vGAT and gephyrin spots in apposition indicative for GABAergic synapses (Fig. 3D), nor after quantification of pre- or postsynaptic markers, separately (Fig. 3E, F). In conclusion, separate cultures of NGN2 or AD2 neurons reveal reduced glutamatergic synapse densities while inhibitory synapses did not show an overt phenotype.

### Disturbed signaling pathways in *DISC1* ± neurons

A highly sensitive, Western-Blot-based high-throughput approach for targeted protein profiling (DigiWest^®^) was applied to verify deregulation of known DISC1-associated signaling pathways in differentiated neurons. Chosen target proteins included synaptic or neuronal markers, and members of well-known signaling pathways, directly or indirectly modulated by DISC1. In accordance, components of the WNT signaling pathway were included which regulate GSK3β activity along with beta-catenin abundance [30]. Likewise, growth factor signaling proteins attributable to the Akt/mTOR, MAPK, PLC-γ and Jak/STAT pathways were examined which are pivotal to the DISC1-dependent regulation of diverse cellular functions such as differentiation and proliferation [31, 32]. In total, expression of a set of 88 proteins and phospho-proteins was studied (Additional file 2: Table S1), of which 68 showed detectable signals. Accumulated fluorescence intensity values (AFI) and normalized values can be found in Additional file 3: Table S2 and Additional file 4: Table S3. Of the 68 analytes with sufficient signals, 8 (~ 11.7%) were significantly deregulated in NGN2 *DISC1* ± neurons and 5 (~ 7.3%) in AD2 *DISC1* ± neurons suggesting differential regulation of proteins in the two populations of neurons (Additional file 1: Figure S7).

**Fig. 4:**
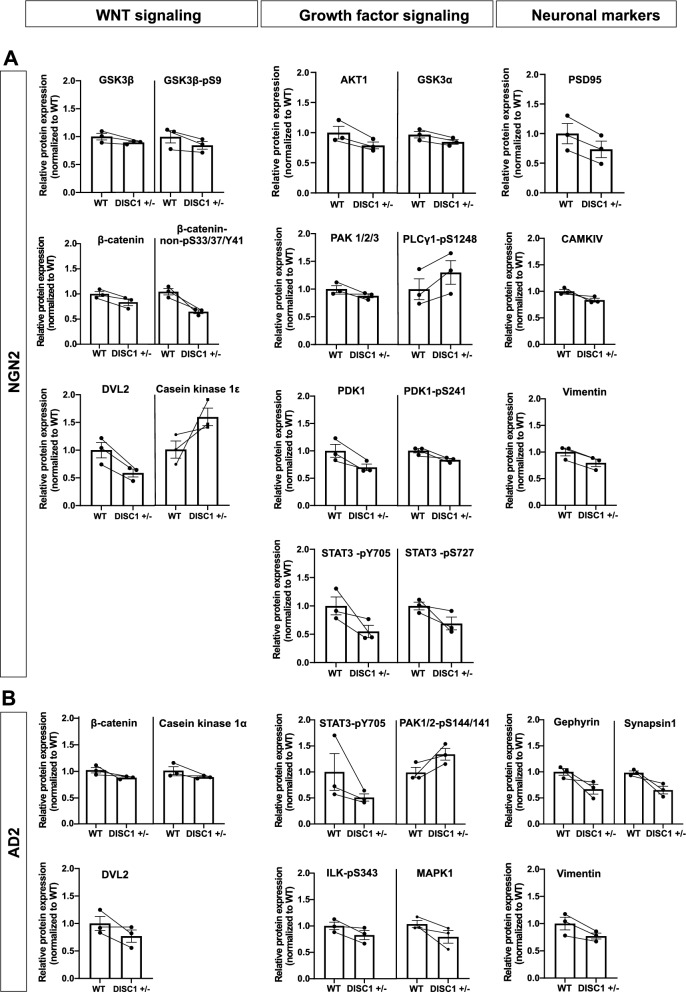
Common and divergent protein profiles in *DISC1* ± NGN2 and AD2 neurons. **A**, **B** Bar chart representations of selected analytes measured in NGN2 (**A**) and AD2 (**B**) neurons. Data were normalized to WT signals within individual replicates, data points from the same replicate were connected. (*DVL2* Segment polarity protein dishevelled homolog DVL-2, *GSK3* Glycogen synthase kinase-3, *PSD95* Postsynaptic density protein 95, *CAMKIV* Calcium/calmodulin-dependent protein kinase type *IV, AKT1* RAC-alpha serine/threonine-protein kinase, *PDK1* phosphoinositide-dependent protein kinase 1, *PLCγ1* Phospholipase C gamma 1, *PAK1/2/3* Serine/threonine-protein kinase PAK 1/2/3, *STAT3* Signal transducer and activator of transcription 3, *ILK1* Integrin-linked protein kinase 1, *MAPK1* mitogen-activated protein kinase 1). Error bars: s.e.m. (NGN2: β-catenin WT = 1 ± 0.09, *DISC1* ±  = 0.8 ± 0.1, DVL2 WT = 1 ± 0.1, *DISC1* ±  = 0.6 ± 0.07, STAT3-pY705 WT = 1 ± 0.16, *DISC1* ±  = 0.55 ± 0.12, Cyclin D2 WT = 1 ± 0.14, *DISC1* ±  = 1.39 ± 0.26, GSK3β WT = 1 ± 0.06, *DISC1* ±  = 0.89 ± 0.02, casein kinase 1ε WT = 1 ± 0.15, *DISC1* ±  = 1.58 ± 0.16, AKT1 WT = 1 $\pm$ 0.1, *DISC1* ±  = 0.79 ± 0.06, PDK1 WT = 1 ± 0.12, *DISC1* ±  = 0.7 ± 0.06, PDK1-pS241 WT = 1 ± 0.04, *DISC1* ±  = 0.83 $\pm$ 0.03, GSK3α WT = 1 ± 0.14, *DISC1* ±  = 0.92 $\pm$ 0.04, PLCγ1–pS1248 WT = 1 ± 0. 19, *DISC1* ±  = 1.3 ± 0.21, PAK1/2/3 WT = 1 ± 0.06, *DISC1* ±  = 0.88 ± 0.04, STAT3-pS272 WT = 1 ± 0.07, *DISC1* ±  = 0.69 ± 0.11; AD2: β-catenin WT = 1 ± 0.05, *DISC1* ±  = 0.85 ± 0.02, DVL2 WT = 1 ± 0.1, *DISC1* ±  = 0.77 ± 0.11, STAT3-pY705 WT = 1 ± 0.35, *DISC1* ±  = 0.51 ± 0.07, Cyclin D2 WT = 1 ± 0.12, *DISC1* ±  = 1.45 ± 0.24, casein kinase 1α WT = 1 ± 0.07, *DISC1* ±  = 0.88 ± 0.02, PAK1/2-pS144/141 WT = 1 ± 0.1, *DISC1* ±  = 1.35 ± 0.11, ILK1-pS343 WT = 1 ± 0.07, *DISC1* ±  = 0.83 ± 0.09, MAPK1 WT = 1 ± 0.07, *DISC1* ±  = 0.77 ± 0.12)

Analytes for which a consistent trend was observed across three independent neuronal differentiations are shown in Fig. 4A, B. Here, deregulated proteins of both neuronal phenotypes fall into the three major groups comprising WNT signaling, growth factor signaling, and neuronal proteins while differences in the expression of single group members do persist. It is of note that β-catenin and deshevelled (DVL2), involved in WNT signaling became deregulated in both NGN2 and AD2 neurons. Likewise, for growth factor signaling, NGN2 and AD2 neurons share deregulated expression of phosphorylated signal transducer and activator of transcription 3 (STAT3-pY705). Notably, expression of cell cycle regulator Cyclin D2 was increased in both NGN2 and AD2 neurons (Additional file 3: Table S2, Additional file 4: Table S3). In contrast, differences in WNT signaling between WT and *DISC1* ± neurons were observed in NGN2 neurons regarding total and phosphorylated glycogen synthase-kinase 3β (GSK3β) and casein kinase 1ε, while casein kinase 1α was more deregulated in AD2 neurons. In the case of growth factor signaling, NGN2 neurons showed deregulated expression of RAC-alpha serine/threonine-protein kinase 1 (AKT1), phosphoinositide-dependent protein kinase 1 (PDK1), PDK1-phophoS241, GSK3α, phosphorylated phospholipase Cγ1 (PLCγ1–pS1248), PAK1/2/3, and STAT3-phosphoS272 while in AD2 neurons expression of phosphorylated PAK1/2-pS144/141, phosphorylated integrin-linked protein kinase 1 (ILK1-pS343), involved in cytokine and growth factor signaling, and mitogen-activated protein kinase 1 (MAPK1) was altered.

Interestingly, postsynaptic scaffolding protein PSD95 found in glutamatergic synapses was downregulated in NGN2 neurons while the postsynaptic scaffolding protein gephyrin was downregulated in AD2 neurons. Intermediate filament component vimentin was downregulated in *DISC1* ± cultures of both cell types. Vimentin is expressed by various cell types in the human central nervous system, including neural progenitor cells, immature neurons and astrocytes [33]. Since no astrocytes were added to the cultures for proteomic analysis and vimentin is replaced by neurofilaments in mature neurons [34], the expression in our cultures can likely be attributed to NPC or less mature neurons.. Differences between NGN2 neurons and AD2 neurons were observed regarding the deregulated expression of calcium/calmodulin dependent protein kinase IV (CAMKIV) and synapsin 1, respectively. In summary, protein profiling reveals that previously identified DISC1-mediated signaling became affected in the *DISC1* ± line.

Thus far, we have assured using a series of different approaches, that the *DISC1* ± line displays known phenotypic characteristics and therefore may serve as a representative cell line to mimic DISC1 deficiency for a comparison of separate cultures of NGN2 and AD2-transduced neurons with a co-culture of both.

### Co-cultures of *DISC1* ± NGN2 and ASCL1/DLX2 neurons reveal E/I imbalance

**Fig. 5:**
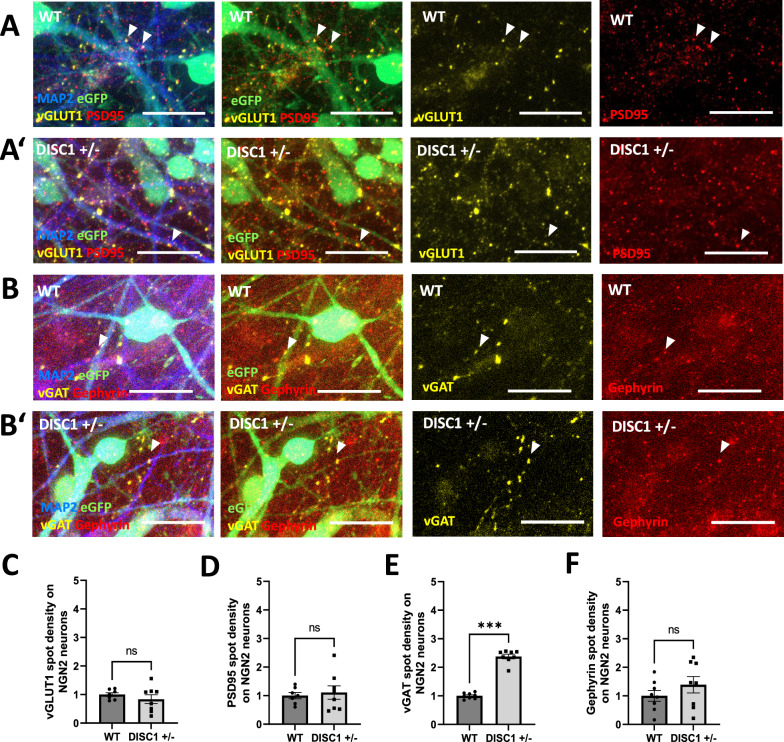
NGN2-AD2 co-culture: Synaptic marker densities in *DISC1* ± NGN2 neurons. **A-A’** Immunostaining for excitatory synapse marker densities on eGFP-positive WT NGN2 neurons (**A**) and *DISC1* ± NGN2 neurons (**A’**). Exemplary co-localizations of pre- and postsynaptic markers are indicated by arrows. Scale bars: 20 µm. **B-B’** Immunostaining for inhibitory synapse markers on eGFP-positive WT NGN2 neurons (**B**) and *DISC1* ± NGN2 neurons (**B’**). Exemplary co-localizations of pre- and postsynaptic markers are indicated by arrows. Scale bars: 20 µm. **C–F** Quantification of glutamatergic and GABAergic synapse marker densities on MAP2/eGFP double-positive dendrites in WT and *DISC1* ± NGN2 neurons. Data were obtained from four independent differentiations. Error bars: s.e.m. (**C**) presynaptic VGLUT1, WT n = 7, *DISC1* ± n = 8, WT = 1 ± 0.07, *DISC1* ±  = 0.84 ± 0.16, Mann-Whitney U test, two-tailed, p = 0.3969 (**D**) postsynaptic PSD95, WT n = 7, *DISC1* ± n = 8, WT = 1 ± 0.12, *DISC1* ±  = 1.11 ± 0.23, Mann-Whitney U test, two-tailed, p = 0.8665 (**E**) presynaptic vGAT, WT n = 8, *DISC1* ± n = 8, WT = 1 ± 0.04, *DISC1* ±  = 2.38 ± 0.08, Mann-Whitney U test, two-tailed, p = 0.0002. **F** postsynaptic gephyrin, WT n = 8, *DISC1* ± n = 8, WT = 1 ± 0.19, *DISC1* ±  = 1.39 ± 0.29, Mann-Whitney U test, two-tailed, p = 0.3823.

**Fig. 6:**
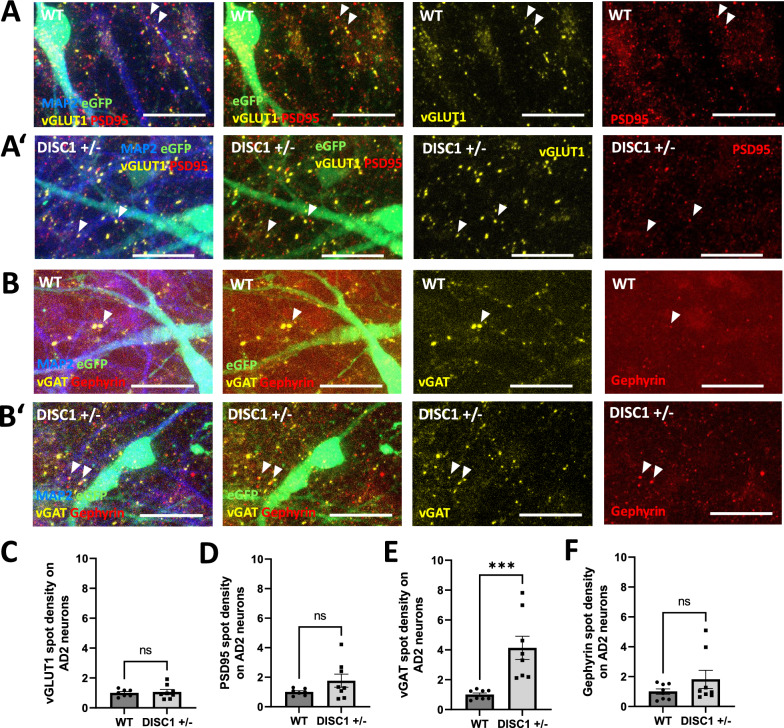
NGN2-AD2 co-culture: Synaptic marker densities in *DISC1* ± AD2 neurons. **A-A’** Immunostaining for excitatory synapse marker densities on MAP2-positive, eGFP-negative WT AD2 neurons (**A**) and *DISC1* ± AD neurons (**A’**). Exemplary co-localizations of pre- and postsynaptic markers are indicated by arrows. Scale bars: 20 µm. **B-B’** Immunostaining for inhibitory synapse marker densities on MAP2-positive, eGFP-negative WT AD2 neurons (**B**) and *DISC1* ± AD2 neurons (**B’**). Exemplary co-localizations of pre- and postsynaptic markers are indicated by arrows. Scale bars: 20 µm. **C–F** Quantification of glutamatergic and GABAergic synapse marker densities on MAP2-positive, eGFP-negative dendrites in WT and *DISC1* ± AD2 neurons. Data were obtained from four independent differentiations. Error bars: s.e.m. (**C**) presynaptic VGLUT1, WT n = 7, *DISC1* ± n = 8, WT = 1 ± 0.09, *DISC1* ±  = 1.07 ± 0.16, Mann–Whitney U test, two-tailed, p = 0.9551 (**D**) postsynaptic PSD95, WT n = 7, *DISC1* ± n = 8, WT = 1 ± 0.09, *DISC1* ±  = 1.77 ± 0.44, Mann–Whitney U test, two-tailed, p = 0.2319.** E** presynaptic vGAT, WT n = 8, *DISC1* ± n = 8, WT = 1 ± 0.11, *DISC1* ±  = 4.13 ± 0.78, Mann–Whitney U test, two-tailed, p = 0.0002. **F** postsynaptic gephyrin WT n = 8, *DISC1* ± n = 8, WT = 1 ± 0.18, *DISC1* ±  = 1.82 ± 0.61, Mann–Whitney U test, two-tailed, p = 0.5737

For modelling the neuronal microcircuitry comprising excitatory and inhibitory neurons in a more defined and potentially more mature culture system, the two populations of NGN2 and AD2 neurons were subsequently mixed at a ratio of 80:20, resembling the approximate ratio of glutamatergic to GABAergic neurons reported in the cortex [35–37]. NGN2-transduced neurons co-expressed eGFP to allow for discrimination from eGFP-negative, AD2-transduced neurons. Pre- and postsynaptic marker expression in conjunction with eGFP-positive and eGFP-negative cells was chosen as an approximation to assign synaptic structures to either NGN2 or AD2 neurons (Figs. 5A, A’, B, B’, 6A, A’, B, B’). Overview images from which the zoomed-in image sections in Figs. 5 and 6 were obtained can be found in Additional file 1: Figures S8 and S9.

Quantification of presynaptic marker vGLUT1 as well as of postsynaptic markers PSD95 and gephyrin on eGFP-positive NGN2 neurons did not show any overt differences between WT and *DISC1* ± neurons (Fig. 5C, D, F). In contrast, presynaptic vGAT spot density on NGN2 neurons was strongly increased by a factor of about 2.4 (Fig. 5E). Subsequently, synaptic marker densities were quantified on eGFP-negative AD2 neurons. Marker densities for glutamatergic input, namely vGLUT1 and PSD95 were not significantly altered on *DISC1* ± AD2 neurons (Fig. 6C, D). However, GABAergic presynaptic input (vGAT) was increased by a factor of ~ fourfold while postsynaptic gephyrin cluster densities were unchanged (Fig. 6E, F).

**Fig. 7:**
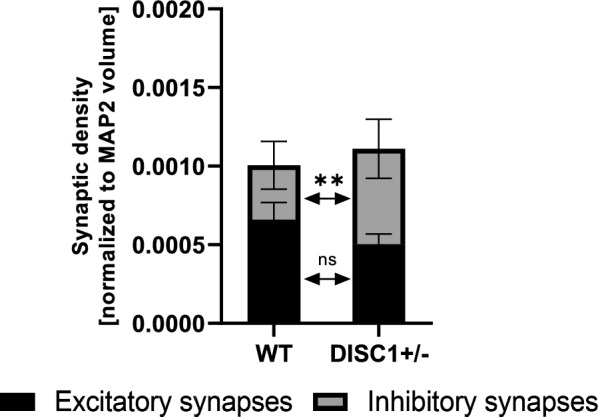
Ratio of excitatory to inhibitory synapses (E/I) in co-cultures of NGN2 and AD2 neurons. **A** Quantification of synaptic densities, as defined by co-localized vGLUT1/PSD95 or vGAT/gephyrin clusters, in the MAP2-positive network of co-cultured NGN2 and AD2 WT and *DISC1* ± neurons. Details on data points and statistics shown in Fig. 7 can be found in Additional file 1: Figure S5C, F. Y-axis values represent synapse marker spot numbers normalized to the MAP2 volume. Error bars: s.e.m

Next, we examined apposition of pre- and postsynaptic markers indicative of morphologically intact excitatory (vGLUT1 + PSD95) or inhibitory (vGAT + gephyrin) synapses (Additional file 1: Figure S10, Fig. 7). Separate analyses of NGN2 and AD2 neurons in co-culture revealed non-significant trends for less excitatory synapses and increased inhibitory synapses on NGN2 neurons (Additional file 1: Figure S10A, D). On AD2 neurons we observed no alterations regarding excitatory synapses (Additional file 1: Figure S10B) as well as slightly increased inhibitory synapses on AD2 neurons (Additional file 1: Figure S10E). However, synapse quantification in the whole MAP2-positive network revealed a significant increase in GABAergic synapse densities and no change in overall excitatory synapse densities (Additional file 1: Figure S10C, F). These alterations of synapse numbers result in a shift of the overall synaptic balance in the MAP2 neuronal network, leading to an E/I imbalance via increased inhibition (Fig. 7). The observed synaptic E/I imbalance is in line with previous findings in a mouse model with reduced DISC1 expression [15].

### Calcium imaging reveals increased activation of *DISC1* ± AD2 neurons

**Fig. 8:**
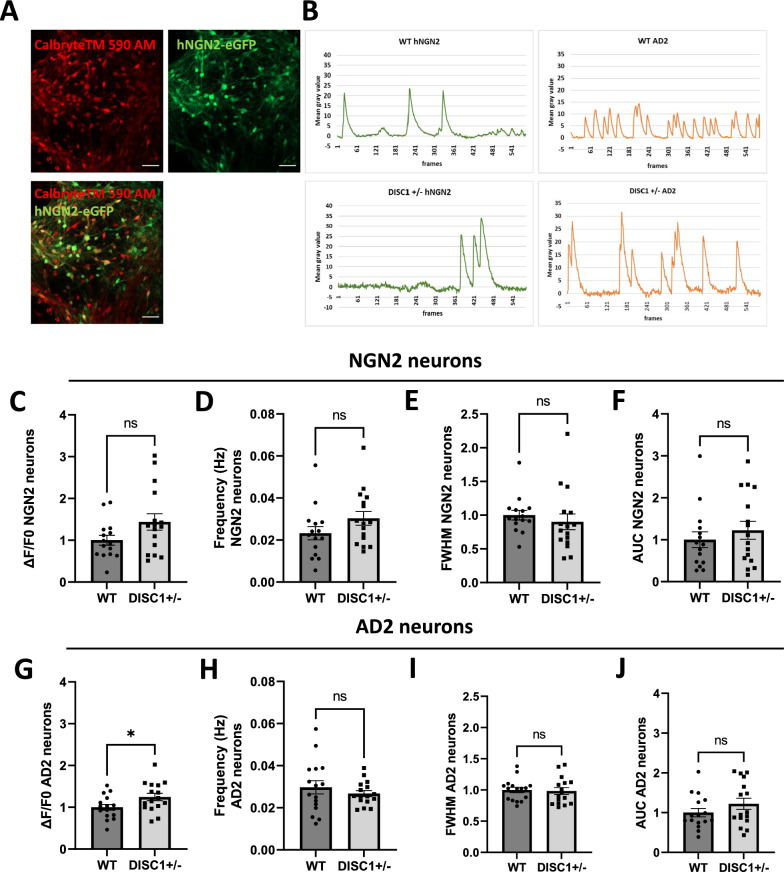
NGN2-AD2 co-culture: Single-cell calcium activity. **A** Exemplary image of DIV28 WT NGN2/AD2 co-cultures. Neurons were stained with Calbryte^™^ 590 AM for analysis of intracellular calcium dynamics. NGN2 (eGFP-Calbryte-double positive) and AD2 neurons (eGFP negative) were examined, separately. Scale bars: 50 µm. **B** Exemplary calcium traces of WT and *DISC1* ± NGN2 and AD2 neurons. Mean gray values of individual cell somata are plotted over a recording period of 3 min (583 frames). **C–F** Somatic calcium trace properties of NGN2 neurons in co-cultures. Peak amplitude = ΔF/F0), frequency (Hz), full width half maximum = FWHM, area under the curve = AUC. Data were retrieved from three independent experiments, with a minimum of three wells recorded per experiment and group (**C**: WT n = 15, *DISC1* ± n = 16, WT = 1 ± 0.12, *DISC1* ±  = 1.44 ± 0.19, Mann Whitney test, two-tailed, p = 0.1195 **D**: WT n = 15, *DISC1* ± n = 16, WT = 0.02 ± 0.003, *DISC1* ±  = 0.03 ± 0.003, Mann Whitney test, two-tailed, p = 0.1122 **E**: WT n = 15, *DISC1* ± n = 16, WT = 1 ± 0.07, *DISC1* ±  = 0.9 ± 0.12, Mann Whitney test, two-tailed, p = 0.0933 **F**: WT n = 15, *DISC1* ± n = 16, WT = 1 ± 0.19, *DISC1* ±  = 1.23 ± 0.21, Mann Whitney test, two-tailed, p = 0.5196). **G-J** Somatic calcium traces retrieved from AD2 neurons in co-cultures. Data were obtained from three independent experiments, with four wells recorded per experiment and condition (**G**: WT n = 16, *DISC1* ± n = 16, WT = 1 ± 0.07, *DISC1* ±  = 1.25 ± 0.09, unpaired t-test, two-tailed, p = 0.0362 **H**: WT n = 16, *DISC1* ± n = 16, WT = 0.029 ± 0.003, *DISC1* ±  = 0.026 ± 0.001, unpaired t-test, two-tailed, p = 0.4002 **I:** WT n = 16, *DISC1* ± n = 16, WT = 1 ± 0.04, *DISC1* ±  = 0.98 ± 0.05, unpaired t-test, two-tailed, p = 0.8103 **J**: WT n = 16, *DISC1* ± n = 16, WT = 1 ± 0.11, *DISC1* ±  = 1.22 ± 0.14, unpaired t-test, two-tailed, p = 0.2189)

In a further set of experiments, we performed calcium imaging to monitor spontaneous neuronal activity in eGFP-positive, NGN2 neurons in comparison to eGFP-negative AD2 neurons. NGN2/AD2 co-cultures were loaded with a red emitting calcium sensor (Calbryte^™^ 590 AM) (Fig. 8A) and calcium signals were recorded for 3 min (Fig. 8B). Calcium signals were measured at the soma of individual neurons, excluding astrocytic calcium signals due to the distinct morphology of the cells. Analysis of calcium signals of parental WT compared to mutated *DISC1* ± neurons showed no changes of calcium parameters in NGN2 neurons (Fig. 8C–F). AD2 neurons showed an increase of peak amplitudes (ΔF/F0), while peak frequency, full-width half-maximum (FWHM) and area under the curve (AUC) were unchanged (Fig. 8G–J). Thus, the more pronounced activity of AD2, mostly inhibitory neurons, as compared to NGN2, mostly excitatory neurons, suggests functional imbalance in *DISC1* ± cultures.

## Discussion

iPSC-derived neurons provide a valuable tool to study disease mechanisms of psychiatric disorders in a relevant human model system. In this paper we aimed at the evaluation of a co-culture model comprising a defined ratio of NGN2 and AD2 neurons. Using DISC1 heterozygous neurons as an example, the co-culture revealed an impact of *DISC1* mutation on GABAergic neurons at the morphological level which cannot be observed with separate cultures comprising either NGN2 or AD2 neurons only. A specific contribution was further confirmed by the observed selective activation of calcium signaling in AD2 neurons. Although it might represent an important mechanism contributing to the pathology of psychiatric disorders [38–40], GABAergic synaptic alterations of *DISC1* mutant neurons were so far only studied in mice but not in models with human iPSC-derived neurons [15].

DISC1 protein was previously shown to be important for neuronal migration, neurite outgrowth and synapse formation. These functions conform with the supposed role of DISC1 as a risk factor for psychiatric diseases associated with a developmental phenotype. Disorganization of neuronal connectivity, especially disbalance between excitation and inhibition at the synaptic level, may play a major role. To elucidate DISC1 function in a human iPSC-based model, we have chosen a frame shift mutation in exon 2 in an isogenic iPSC line to remove most of the DISC1 coding sequence and to exploit haploinsufficiency after COOH-terminal truncation for reduced DISC1 expression [22]. Accordingly, quantitative immunocytochemistry with an antibody specific for the COOH-terminus of DISC1 revealed decreased DISC1 expression. Likewise, the observed decline in neurite outgrowth of the *DISC1* ± clone is in agreement with several reports using different experimental approaches: RNA interference with DISC1 or overexpression of dominant-negative variants of the DISC1 interactors NDEL1 or FEZ1 reduce neurite outgrowth [23, 41]. Likewise, overexpression of a DISC1 variant truncated at the carboxyterminus impaired neurite outgrowth of PC12 cells [24, 42]. So far, only one report analyzed neurite extension in an iPSC-based mutant DISC1 model with a 4 bp insertion at the COOH-terminus [3]. This report indicated increased neurite outgrowth, while other parameters such as synapse formation and decline in presynaptic release point at an impaired rather than improved differentiation.

The divergent results in the NGN2/AD2 co-culture model as compared to cultures with only one neuronal type suggest an interaction of the two populations of neurons, contributing to the reported GABAergic phenotypes in *DISC1* ± cultures. We observed enhanced differentiation efficiency of *DISC1* ± AD2-transduced neurons towards the GABAergic lineage, which could contribute to the increase of GABAergic synaptic markers in co-cultures. Since this phenotype was not detected in separate cultures of AD2 neurons, the interaction with primarily glutamatergic NGN2 neurons in the co-cultures during the maturation period could exacerbate this intrinsic phenotype of AD2 neurons, resulting in excess formation of presynaptic terminals. In rodent models, the possibility of such an interaction has been previously documented after overexpression of mutant *DISC1* in excitatory cortical neurons that decreased the density of parvalbumin-positive interneurons demonstrating a mechanism of how excitatory neurons can modulate the differentiation of interneurons, indirectly [43]. In a further report, decreased DISC1 expression in immature granule cells, the principal neurons of the dentate gyrus, was shown to elicit increased GABAergic synapse formation [15]. In a third study, knockdown of *DISC1* in interneurons accounted for increased inhibitory GAD65-positive synaptic terminals onto pyramidal neurons [14]. Hence, mutant *DISC1*, both in excitatory and inhibitory neurons, increases inhibitory input onto excitatory neurons. Additionally, in the two latter reports, increased frequency of either miniature excitatory postsynaptic currents in interneurons or increased GABAergic spontaneous synaptic currents in excitatory neurons were observed, respectively. Taken together, this agrees with our findings in our human co-culture model where we found increased inhibitory presynaptic input onto NGN2 neurons and increased calcium signaling of AD2 neurons as an indication for enhanced GABAergic drive.

One limitation of our study is that we cannot exclude the presence of other neuronal cell types in NGN2 and AD2-transduced cultures. Our results suggest that while GABAergic neurons are almost absent in NGN2 cultures, only ~ 50% of neurons in AD2 cultures are GABA positive. While previous studies report high efficiency of GABAergic conversion of iPSC (~ 90% GABA-positive neurons) [10], the percentage of resulting GABA-positive cells has not been reported for AD2 transduction of NPC, which we chose as a starting cell type. To this end, it is important to optimize the differentiation protocol or prolong the cultivation period to obtain a more homogeneous population of GABAergic neurons in future experiments. Nevertheless, co-cultivation of NGN2 and AD2 transduced neurons could improve the maturity of the resultant cultures as suggested by previous reports [7, 10].

## Conclusions

In the present study, we used a pair of isogenic iPSC lines, one with a heterozygous mutation in *DISC1*, to investigate phenotypes of mutant NGN2 and AD2-transduced neurons. Initial experiments with NGN2 neurons cultured separately confirmed known DISC1-associated phenotypes, namely reduced excitatory synapse numbers and reduced neurite outgrowth, while inhibitory synapses of AD2 neurons remained unaffected. Targeted proteomic analysis revealed shared deregulation of proteins involved in Wnt signaling, growth factor signaling, and of neuronal proteins in both types of neurons. In co-cultures of NGN2 and AD2 neurons, we observed a strong GABAergic phenotype, which has not been reported before in an iPSC model. An overall increase in inhibitory synapses evoked a shift in E/I balance, which was supported by the finding of increased neuronal activity specifically in AD2 neurons. Overall, the NGN2/AD2 neuron co-culture model can serve as a suitable system to study early morphological and functional phenotypes of neuropsychiatric diseases in human iPSC-derived neurons. Our findings of aberrant phenotypes in GABAergic neurons provide an additional perspective for deciphering the role of DISC1 in neurodevelopmental disorders.

### Supplementary Information

**Additional file1: Figure S1.** Synapse analysis workflow with IMARIS software. Confocal microscopy z-stack images are individually analyzed using IMARIS (Oxford Instruments) to quantify presynaptic and postsynaptic densities on neuronal dendrites and somata. To analyze all synapses in the neuronal network, synaptic signals were assigned to a mask of MAP2-positive neurons, while synaptic markers were detected within a mask of eGFP-positive neurons to inform on synaptic signal densities on NGN2 neurons. Synapse densities on AD2 neurons were retrieved by subtraction of both values. For a detailed description of the IMARIS workflow, see ‘Imaging and analysis of synaptic marker expression’ in the Methods section. **Figure S2.** DigiWest Workflow. **A** SDS-PAGE. **B** Western blot and biotinylation of proteins with subsequent cutting of each lane into 96 stripes representing molecular mass fractions. **C** Elution of proteins from single membrane sections molecular mass protein fractions into 96-well plates. **D** Protein loading onto magnetic color-coded neutravidin-coated Luminex^®^ beads. **E** Pooling of beads. **F** Bead mixes become incubated with predefined mixtures of primary antibodies to give yield to multiplexed antibody-based immunoassays. **G** The Luminex^®^ instrument measures immunoassay signal intensities in the context of color-coded beads indicative for a molecular mass fraction. Integrated signal intensities are represented as a digital Western Blot. **H** DigiWest protein profile derived from digital Western Blot signals.** Figure S3.** Quantification of DISC1 protein levels by western blot. **A **Western blot showing DISC1 protein expression in WT and *DISC1+/−* iPSC, using an antibody directed against the N-terminus of the protein. Beta-actin (42 kDa) was used as a loading control. The three lanes for each clone correspond to three individual protein samples isolated from iPSC (three independent replicates). A DISC1 band was detected at $\sim$95 kDa, corresponding to the L/Lv-isoform of the DISC1 protein. However, several further bands at varying smaller molecular weights were detected as well, which might correspond to additional DISC1 isoforms or could be due to unspecific antibody binding, a problem that has been described for multiple commercially available DISC1 antibodies by Kuroda et al. The blot was cropped to exclude additional samples not included in this study. **B** Quantification of relative DISC1 protein expression (95 kDa band). Data was normalized to beta-actin and WT mean. **Figure S4.** NPC proliferation and cell death. **A **Unbiased, automatized analysis of NPC proliferation measured every 4 h over a period of 72 h shows increased proliferation of *DISC1+/−* NPC at t=68 h and t=72 h. Data were obtained from three independent experiments with at least 5 wells measured per experiment and group (2way ANOVA with Šídák's multiple comparisons test, t= 68 h: WT = 2.02$\pm$0.27, *DISC1+/−* = 2.82$\pm$0.32, p=0.0193; t= 72 h: WT = 2.12$\pm$0.31, *DISC1+/−* = 2.96$\pm$0.35, p= 0.0136). n=20 wells (**B**) Cell death of NPC was measured every 4 h over a period of 72 h and is unaltered in *DISC1 +/−* NPC. Data were obtained from 3 independent experiments with at least 5 wells measured per experiment and group (2way ANOVA with Šídák's multiple comparisons test). n=3 replicates. **Figure S5.** Apposition of pre- and postsynaptic markers in NGN2 and AD2 monocultures. **A** Arrows indicate exemplary co-localizing vGLUT1 and PSD95 spots on MAP2-positive dendrites of NGN2 neurons. **B **Arrows indicate exemplary co-localizing vGAT and Gephyrin spots on MAP2-positive dendrites of AD2 neurons. Scale bars: 20 µm.** Figure S6.** Quantification of GABA-positive cells in NGN2 and AD2 monocultures.** A **Immunocytochemical staining of GABA in WT and *DISC1+/−* NGN2 and AD2 monocultures. Scale bars: 50 µm. **B** Quantification of GABA/MAP2-double positive cells in WT and *DISC1+/−* NGN2 and AD2 cultures from two independent neuronal differentiations. Data points represent values obtained from individual images. Up to 10 cells were analyzed per image. (NGN2 WT: n=20, WT AD2: n= 23, *DISC1+/−* NGN2: n= 20, *DISC1+/−* AD2: n= 19; WT NGN2= 2.33$\pm$1.11, WT AD2= 42.31$\pm$4.18, *DISC1+/−* NGN2= 5.32$\pm$2.09, *DISC1+/−* AD2= 60.89$\pm$5.46, Kruskal-Wallis test with Dunn’s multiple comparisons test, p< 0.0001). Error bars: s.e.m (**C**) Comparison of the percentage of GABA-positive neurons in WT and *DISC1+/−* AD2 monocultures. Data points represent values obtained from individual images. Up to 10 cells were analyzed per image. (WT: n= 23, *DISC1+/−:* n= 19, WT= 42.31$\pm$4.18, *DISC1+/−=* 60.89$\pm$5.46, unpaired t-test, p<0.01).** Figure S7.** Deregulated proteins in NGN2 and AD2 neurons. **A**, **B **Hierarchical cluster analysis of proteins significantly different between WT and *DISC1+/−* DIV 28 NGN2 and AD2 neurons (Wilcoxon test, p< 0.05). For heatmap generation, AFI (accumulated fluorescence intensity) values were median-centered across all samples for a given analyte and Log2-transformed. (*DVL2* Segment polarity protein dishevelled homolog DVL-2, *PDK1* phosphoinositide-dependent protein kinase 1, *CAMKIV* Calcium/calmodulin-dependent protein kinase type IV, *STAT3* Signal transducer and activator of transcription 3, *MAPK1* mitogen-activated protein kinase 1)**. Figure S8.** Expression of synaptic markers in NGN2/AD2 co-cultures as presented in Figure 5. **A-A’ **Immunostaining for excitatory presynaptic marker vGLUT1 and postsynaptic marker PSD95 on MAP2-positive WT (**A**) and *DISC1+/−* (**A’**) neurons. NGN2 neurons in the co-culture express eGFP. Scale bars: 20 µm. **B-B’** Immunostaining for inhibitory presynaptic marker vGAT and postsynaptic marker Gephyrin on MAP2-positive WT (**B**) and *DISC1+/−* (**B’**) neurons. NGN2 neurons in the co-culture express eGFP. Scale bars: 20 µm. Zoomed-in sections of the images can be found in **Figure 5. Figure S9.** Expression of synaptic markers in NGN2/AD2 co-cultures as presented in Figure 6.** A-A’** Immunostaining for excitatory presynaptic marker vGLUT1 and postsynaptic. marker PSD95 on MAP2-positive WT (**A**) and *DISC1+/−* (**A’**) neurons. NGN2 neurons in the co-culture express eGFP. Scale bars: 20 µm. **B-B’** Immunostaining for inhibitory presynaptic marker vGAT and postsynaptic marker Gephyrin on MAP2-positive WT (**B**) and *DISC1+/−* (**B’**) neurons. NGN2 neurons in the co-culture express eGFP. Scale bars: 20 µm. Zoomed-in sections of the images can be found in **Figure 6. Figure S10.** Excitatory and inhibitory synapse densities on co-cultured NGN2 and AD2 neurons.** A-F **Density of excitatory and inhibitory synapses, as defined by co-localization of vGLUT1+PSD95 and vGAT+gephyrin, respectively, in DIV28 WT and *DISC1+/* neurons. Data points represent averaged synapse densities from multiple microscopic fields within one well. Data were obtained from four independent experiments.** A** Excitatory synapse density on NGN2 neurons. WT n=7, *DISC1+/−* n=8, WT = 1$\pm$0.11, *DISC1+/−* = 0.62$\pm$0.11, Mann Whitney U test, two-tailed, p=0.0721. **B** Excitatory synapse density on AD2 neurons. WT n=7, *DISC1+/−* n=8, WT = 1$\pm$0.18, DISC1+/− = 0.94$\pm$0.16, Mann Whitney U test, two-tailed, p=0.6943. **C** Excitatory synapse density on all MAP2-positive neurons. WT n=7, *DISC1+/−* n=8, WT = 1$\pm$0.16, *DISC1+/−* = 0.73$\pm$0.08, Mann Whitney U test, two-tailed, p=0.1893. **D** Inhibitory synapse density (vGAT+gephyrin) on NGN2 neurons. WT n=6, *DISC1+/−* n=6, WT = 1$\pm$0.21, *DISC1+/−* = 2.2$\pm$0.45, Mann Whitney U test, two-tailed, p=0.0931. **E** Inhibitory synapse density (vGAT+gephyrin) on AD2 neurons. WT n=6, *DISC1+/−* n=6, WT = 1$\pm$0.15, *DISC1+/−* = 1.91$\pm$0.45, Mann Whitney U test, two-tailed, p=0.0931. **F** Inhibitory synapse density on all MAP2-positive neurons. WT n=6, *DISC1+/−* n=6, WT = 1$\pm$0.13, *DISC1+/−* = 2.08$\pm$0.42, Mann Whitney U test, two-tailed, p=0.0087.

**Additional file2 Table S1.** Complete list of all primary antibodies used for Digi West proteomic analysis of WT and DISC1+/− neurons.

**Additional file3 Table S2.** List of raw accumulated fluorescence intensity values (AFI) of proteins measured with DigiWest. Analytes with empty cells showed weak and/or unreliable signals and were excluded from detailed analysis (weak = AFI < 49).

**Additional file 4: Table S3.** List of accumulated fluorescence intensity values (AFI) of proteins measured with DigiWest, normalized to total protein (Strep-PE) signal. Analytes with empty cells showed weak and/or unreliable signals and were excluded from detailed analysis (weak = AFI < 49).

## Data Availability

The datasets used and/or analysed during the current study are available from the corresponding author on reasonable request.
