# Supplementary material for: Defined co-cultures of glutamatergic and GABAergic neurons with a mutation in DISC1 reveal aberrant phenotypes in GABAergic neurons
Source: BMC Neurosci. 2024 Mar 4;25:12. doi: 10.1186/s12868-024-00858-z (PMC10910844; doi:10.1186/s12868-024-00858-z)
Supplement: Supplementary file 1 — Additional file1: Figure S1. Synapse analysis workflow with IMARIS software. Confocal microscopy z-stack images are individually analyzed using IMARIS (Oxford Instruments) to quantify presynaptic and postsynaptic densities on neuronal dendrites and somata. To analyze all synapses in the neuronal network, synaptic signals were assigned to a mask of MAP2-positive neurons, while synaptic markers were detected within a mask of eGFP-positive neurons to inform on synaptic signal densities on NGN2 neurons. Synapse densities on AD2 neurons were retrieved by subtraction of both values. For a detailed description of the IMARIS workflow, see ‘Imaging and analysis of synaptic marker expression’ in the Methods section. Figure S2. DigiWest Workflow. A SDS-PAGE. B Western blot and biotinylation of proteins with subsequent cutting of each lane into 96 stripes representing molecular mass fractions. C Elution of proteins from single membrane sections molecular mass protein fractions into 96-well plates. D Protein loading onto magnetic color-coded neutravidin-coated Luminex® beads. E Pooling of beads. F Bead mixes become incubated with predefined mixtures of primary antibodies to give yield to multiplexed antibody-based immunoassays. G The Luminex® instrument measures immunoassay signal intensities in the context of color-coded beads indicative for a molecular mass fraction. Integrated signal intensities are represented as a digital Western Blot. H DigiWest protein profile derived from digital Western Blot signals. Figure S3. Quantification of DISC1 protein levels by western blot. A Western blot showing DISC1 protein expression in WT and DISC1+/− iPSC, using an antibody directed against the N-terminus of the protein. Beta-actin (42 kDa) was used as a loading control. The three lanes for each clone correspond to three individual protein samples isolated from iPSC (three independent replicates). A DISC1 band was detected at [file 12868_2024_858_MOESM1_ESM.docx]

Additional Material

# Figure S1: Synapse analysis workflow with IMARIS software


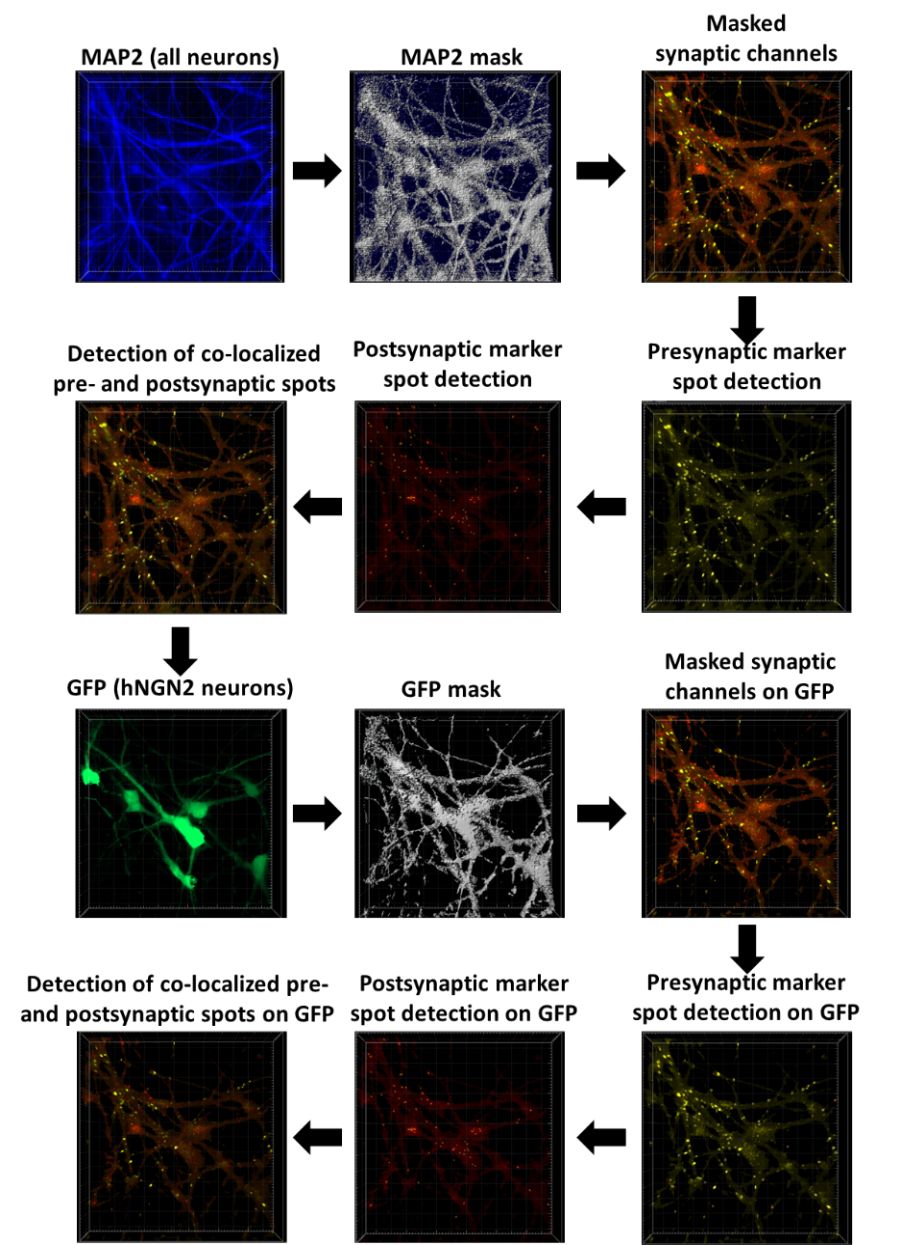


**Figure S1.** Confocal microscopy z-stack images are individually analyzed using IMARIS (Oxford Instruments) to quantify presynaptic and postsynaptic densities on neuronal dendrites and somata. To analyze all synapses in the neuronal network, synaptic signals were assigned to a mask of MAP2-positive neurons, while synaptic markers were detected within a mask of eGFP-positive neurons to inform on synaptic signal densities on NGN2 neurons. Synapse densities on AD2 neurons were retrieved by subtraction of both values. For a detailed description of the IMARIS workflow, see ‘Imaging and analysis of synaptic marker expression’ in the Methods section.

# Figure S2: DigiWest Workflow


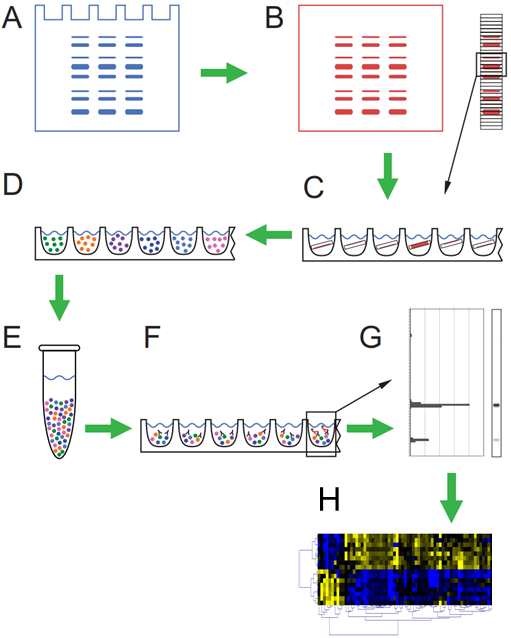


**Figure S2**. **(A)** SDS-PAGE. **(B)** Western blot and biotinylation of proteins with subsequent cutting of each lane into 96 stripes representing molecular mass fractions. **(C)** Elution of proteins from single membrane sections molecular mass protein fractions into 96-well plates. **(D)** Protein loading onto magnetic color-coded neutravidin-coated Luminex® beads. **(E)** Pooling of beads. **(F)** Bead mixes become incubated with predefined mixtures of primary antibodies to give yield to multiplexed antibody-based immunoassays. **(G)** The Luminex® instrument measures immunoassay signal intensities in the context of color-coded beads indicative for a molecular mass fraction. Integrated signal intensities are represented as a digital Western Blot. **(H)** DigiWest protein profile derived from digital Western Blot signals.

**Figure S3: Quantification of DISC1 protein levels by western blot**

**
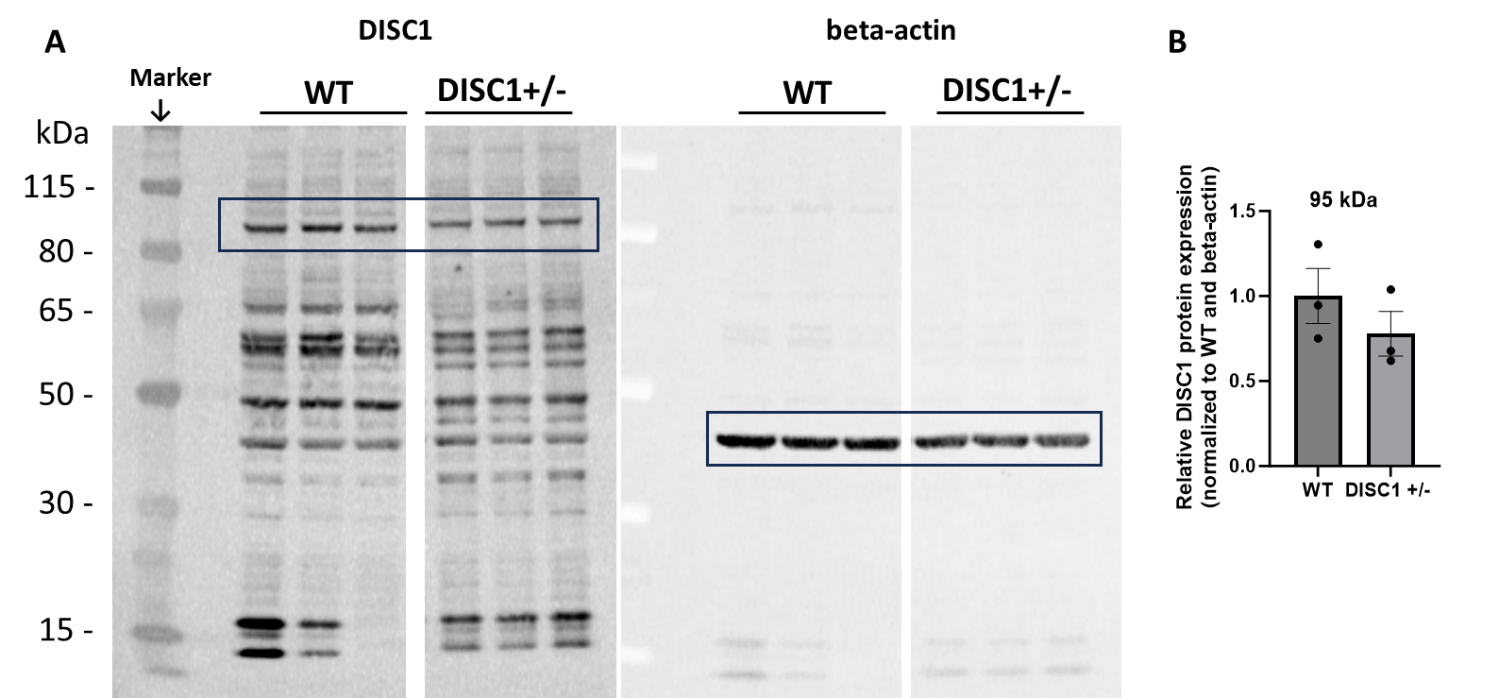
**

**Figure S3. (A)** Western blot showing DISC1 protein expression in WT and *DISC1+/-* iPSC, using an antibody directed against the N-terminus of the protein. Beta-actin (42 kDa) was used as a loading control. The three lanes for each clone correspond to three individual protein samples isolated from iPSC (three independent replicates). A DISC1 band was detected at $\sim$95 kDa, corresponding to the L/Lv-isoform of the DISC1 protein. However, several further bands at varying smaller molecular weights were detected as well, which might correspond to additional DISC1 isoforms or could be due to unspecific antibody binding, a problem that has been described for multiple commercially available DISC1 antibodies by Kuroda et al. (1). The blot was cropped to exclude additional samples not included in this study. (**B)** Quantification of relative DISC1 protein expression (95 kDa band). Data was normalized to beta-actin and WT mean.

# Figure S4: NPC proliferation and cell death

**A**

**B**

**Figure S4. (A)** Unbiased, automatized analysis of NPC proliferation measured every 4 h over a period of 72 h shows increased proliferation of *DISC1+/-* NPC at t=68 h and t=72 h. Data were obtained from three independent experiments with at least 5 wells measured per experiment and group (2way ANOVA with Šídák's multiple comparisons test, t= 68 h: WT = 2.02$\pm$0.27, *DISC1+/-* = 2.82$\pm$0.32, p=0.0193; t= 72 h: WT = 2.12$\pm$0.31, *DISC1+/-* = 2.96$\pm$0.35, p= 0.0136). n=20 wells (**B)** Cell death of NPC was measured every 4 h over a period of 72 h and is unaltered in *DISC1 +/-* NPC. Data were obtained from 3 independent experiments with at least 5 wells measured per experiment and group (2way ANOVA with Šídák's multiple comparisons test). n=3 replicates

# Figure S5: Apposition of pre- and postsynaptic markers in NGN2 and AD2 monocultures


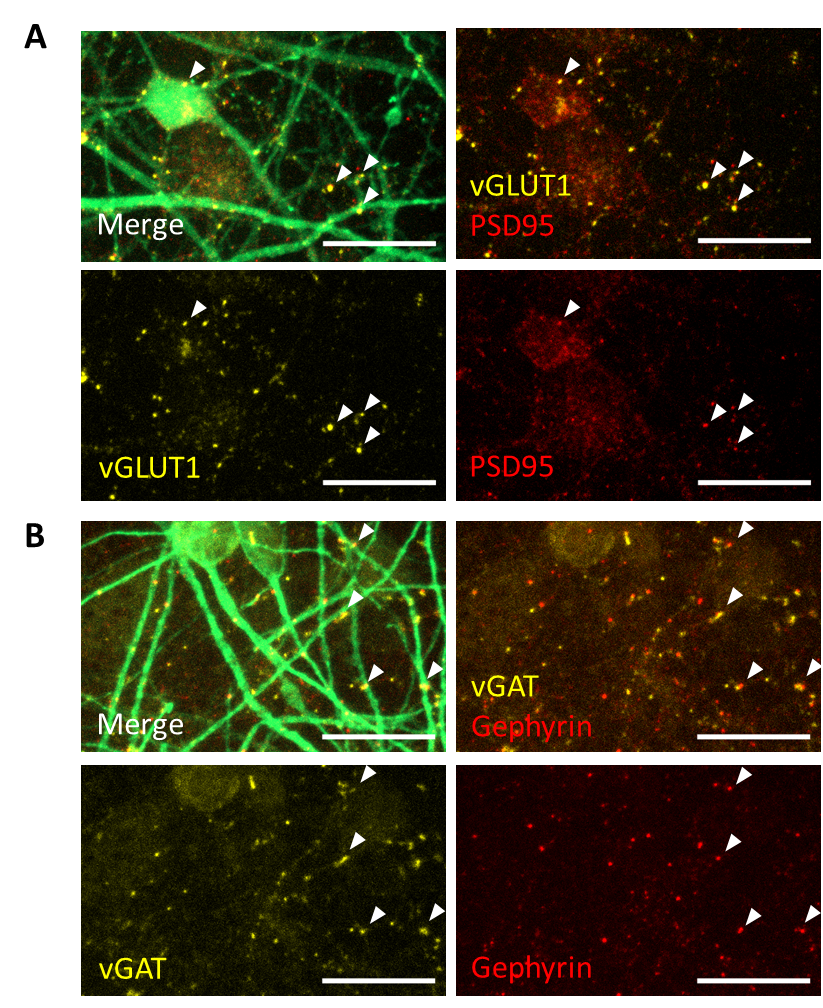


**Figure S5. (A)** Arrows indicate exemplary co-localizing vGLUT1 and PSD95 spots on MAP2-positive dendrites of NGN2 neurons. **(B)** Arrows indicate exemplary co-localizing vGAT and Gephyrin spots on MAP2-positive dendrites of AD2 neurons. Scale bars: 20 µm.

**Figure S6. Quantification of GABA-positive cells in NGN2 and AD2 monocultures**

**
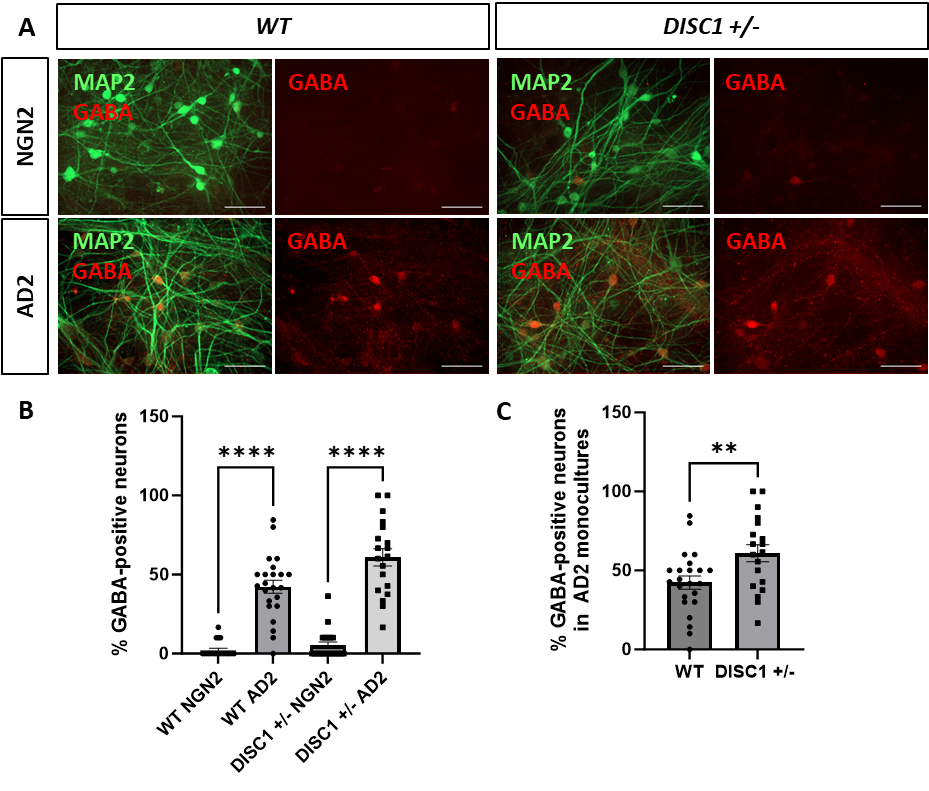
**

**Figure S6. (A)** Immunocytochemical staining of GABA in WT and *DISC1+/-* NGN2 and AD2 monocultures. Scale bars: 50 µm. **(B)** Quantification of GABA/MAP2-double positive cells in WT and *DISC1+/-* NGN2 and AD2 cultures from two independent neuronal differentiations. Data points represent values obtained from individual images. Up to 10 cells were analyzed per image. (NGN2 WT: n=20, WT AD2: n= 23, *DISC1+/-* NGN2: n= 20, *DISC1+/-* AD2: n= 19; WT NGN2= 2.33$\pm$1.11, WT AD2= 42.31$\pm$4.18, *DISC1+/-* NGN2= 5.32$\pm$2.09, *DISC1+/-* AD2= 60.89$\pm$5.46, Kruskal-Wallis test with Dunn’s multiple comparisons test, p< 0.0001). Error bars: s.e.m **(C)** Comparison of the percentage of GABA-positive neurons in WT and *DISC1+/-* AD2 monocultures. Data points represent values obtained from individual images. Up to 10 cells were analyzed per image. (WT: n= 23, *DISC1+/-:* n= 19, WT= 42.31$\pm$4.18, *DISC1+/-=* 60.89$\pm$5.46, unpaired t-test, p<0.01).

**Figure S7: Deregulated proteins in NGN2 and AD2 neurons**


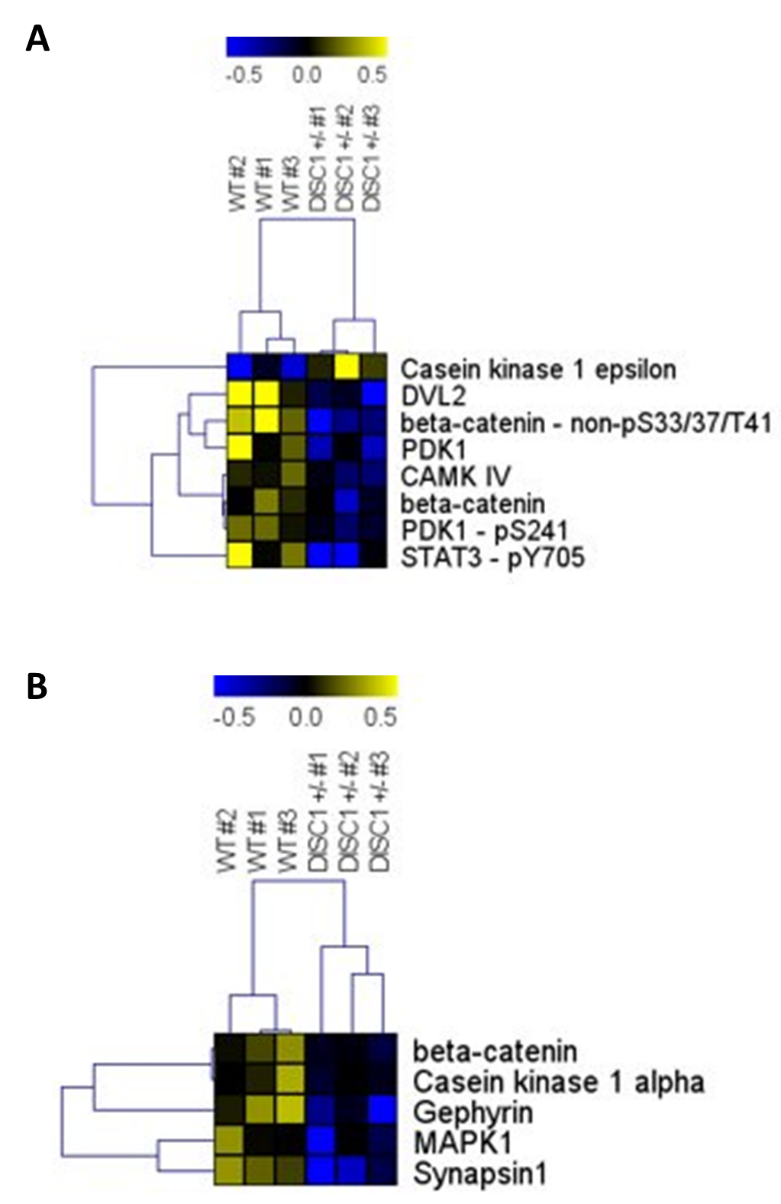


**Figure S7. (A), (B)** Hierarchical cluster analysis of proteins significantly different between WT and *DISC1+/-* DIV 28 NGN2 and AD2 neurons (Wilcoxon test, p< 0.05). For heatmap generation, AFI (accumulated fluorescence intensity) values were median-centered across all samples for a given analyte and Log2-transformed. (DVL2= Segment polarity protein dishevelled homolog DVL-2, PDK1= phosphoinositide-dependent protein kinase 1, CAMKIV= Calcium/calmodulin-dependent protein kinase type IV, STAT3= Signal transducer and activator of transcription 3, MAPK1= mitogen-activated protein kinase 1)

**Figure S8: Expression of synaptic markers in NGN2/AD2 co-cultures as presented in Figure 5.**

**
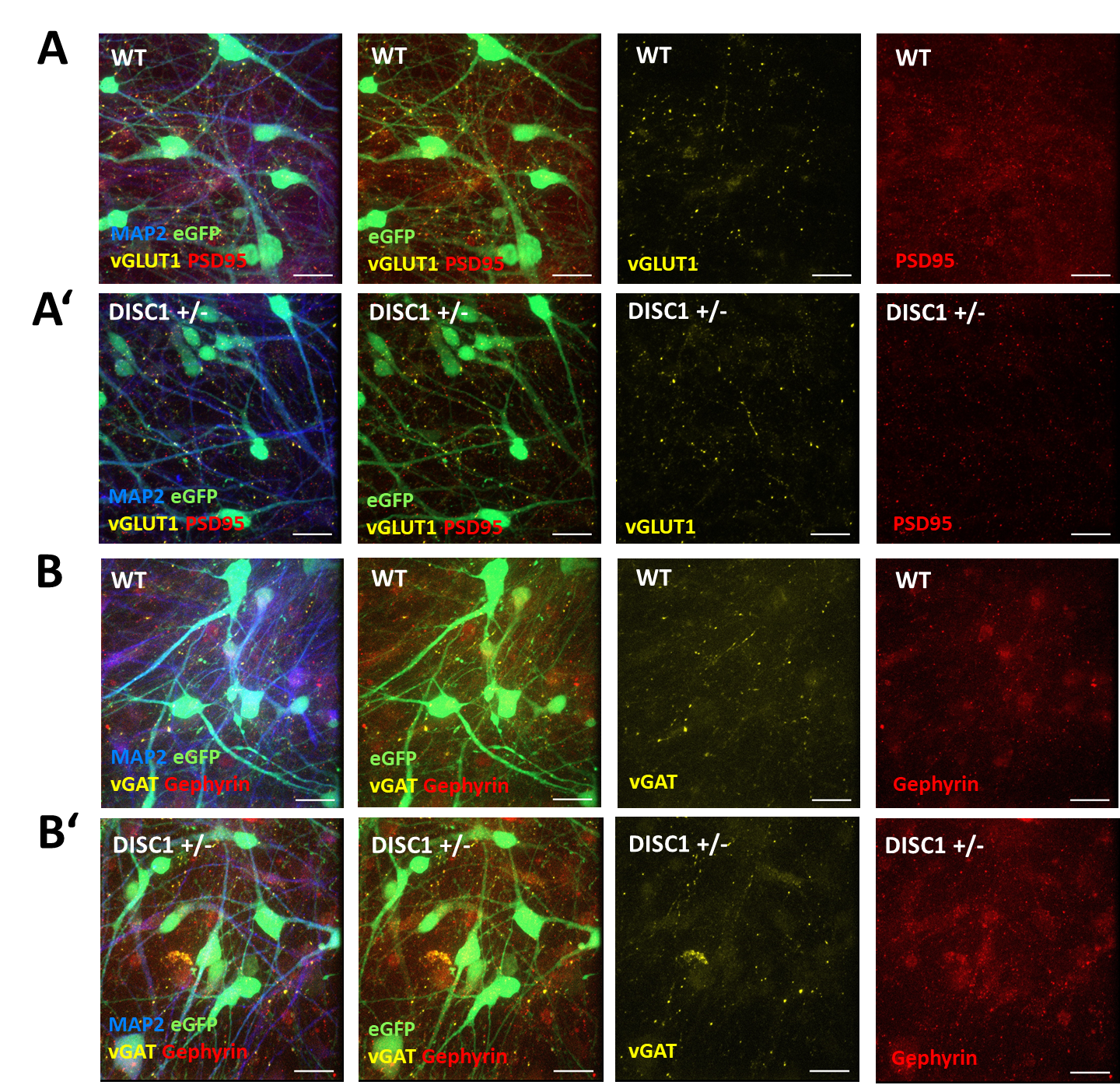
 Figure S8. (A)-(A’)** Immunostaining for excitatory presynaptic marker vGLUT1 and postsynaptic marker PSD95 on MAP2-positive WT **(A)** and *DISC1+/-* **(A’)** neurons. NGN2 neurons in the co-culture express eGFP. Scale bars: 20 µm. **(B)-(B’)** Immunostaining for inhibitory presynaptic marker vGAT and postsynaptic marker Gephyrin on MAP2-positive WT **(B)** and *DISC1+/-* **(B’)** neurons. NGN2 neurons in the co-culture express eGFP. Scale bars: 20 µm. Zoomed-in sections of the images can be found in **Figure 5.**

**
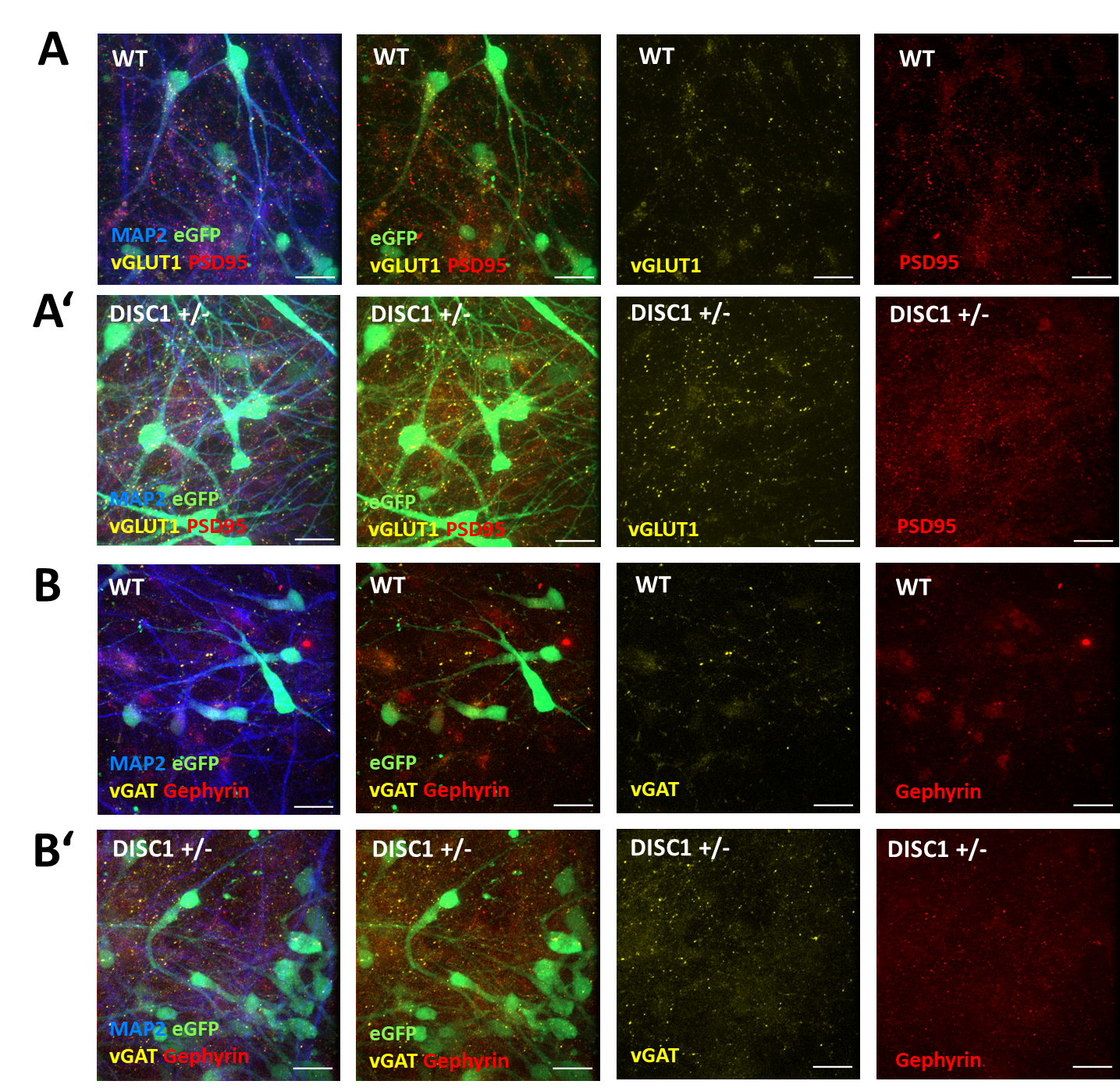
Figure S9: Expression of synaptic markers in NGN2/AD2 co-cultures as presented in Figure 6.**

**Figure S9. (A)-(A’)** Immunostaining for excitatory presynaptic marker vGLUT1 and postsynaptic marker PSD95 on MAP2-positive WT **(A)** and *DISC1+/-* **(A’)** neurons. NGN2 neurons in the co-culture express eGFP. Scale bars: 20 µm. **(B)-(B’)** Immunostaining for inhibitory presynaptic marker vGAT and postsynaptic marker Gephyrin on MAP2-positive WT **(B)** and *DISC1+/-* **(B’)** neurons. NGN2 neurons in the co-culture express eGFP. Scale bars: 20 µm. Zoomed-in sections of the images can be found in **Figure 6.**

**Figure S10: Excitatory and inhibitory synapse densities on co-cultured NGN2 and AD2 neurons.**

**Figure S10. (A)-(F)** Density of excitatory and inhibitory synapses, as defined by co-localization of vGLUT1+PSD95 and vGAT+gephyrin, respectively, in DIV28 WT and *DISC1+/* neurons. Data points represent averaged synapse densities from multiple microscopic fields within one well. Data were obtained from four independent experiments. **(A)** Excitatory synapse density on NGN2 neurons. WT n=7, *DISC1+/-* n=8, WT = 1$\pm$0.11, *DISC1+/-* = 0.62$\pm$0.11, Mann Whitney U test, two-tailed, p=0.0721. (**B)** Excitatory synapse density on AD2 neurons. WT n=7, *DISC1+/-* n=8, WT = 1$\pm$0.18, DISC1+/- = 0.94$\pm$0.16, Mann Whitney U test, two-tailed, p=0.6943. **(C)** Excitatory synapse density on all MAP2-positive neurons. WT n=7, *DISC1+/-* n=8, WT = 1$\pm$0.16, *DISC1+/-* = 0.73$\pm$0.08, Mann Whitney U test, two-tailed, p=0.1893. (**D)** Inhibitory synapse density (vGAT+gephyrin) on NGN2 neurons. WT n=6, *DISC1+/-* n=6, WT = 1$\pm$0.21, *DISC1+/-* = 2.2$\pm$0.45, Mann Whitney U test, two-tailed, p=0.0931. (**E)** Inhibitory synapse density (vGAT+gephyrin) on AD2 neurons. WT n=6, *DISC1+/-* n=6, WT = 1$\pm$0.15, *DISC1+/-* = 1.91$\pm$0.45, Mann Whitney U test, two-tailed, p=0.0931. **(F)** Inhibitory synapse density on all MAP2-positive neurons. WT n=6, *DISC1+/-* n=6, WT = 1$\pm$0.13, *DISC1+/-* = 2.08$\pm$0.42, Mann Whitney U test, two-tailed, p=0.0087.

**References**

1. Kuroda K, Yamada S, Tanaka M, Iizuka M, Yano H, Mori D, et al. Behavioral alterations associated with targeted disruption of exons 2 and 3 of the Disc1 gene in the mouse. Human Molecular Genetics. 2011;20(23):4666-83.
